# Appendicular skeleton of *Protoceratops andrewsi* (Dinosauria, Ornithischia): comparative morphology, ontogenetic changes, and the implications for non-ceratopsid ceratopsian locomotion

**DOI:** 10.7717/peerj.7324

**Published:** 2019-07-22

**Authors:** Justyna Słowiak, Victor S. Tereshchenko, Łucja Fostowicz-Frelik

**Affiliations:** 1 Department of Evolutionary Paleobiology, Institute of Paleobiology, Polish Academy of Sciences, Warsaw, Poland; 2 Laboratory of Paleoherpetology, Paleontological Institute, Russian Academy of Sciences, Moscow, Russia; 3 Key Laboratory of Vertebrate Evolution and Human Origins, Institute of Vertebrate Paleontology and Paleoanthropology, Chinese Academy of Sciences, Beijing, China

**Keywords:** Ceratopsian dinosaurs, Limb morphology, Locomotion, Ornithischia, Ontogeny, Stance

## Abstract

*Protoceratops andrewsi* is a well-known ceratopsian dinosaur from the Djadokhta Formation (Upper Cretaceous, Mongolia). Since the 1920s, numerous skeletons of different ontogenetic stages from hatchlings to adults, including fully articulated specimens, have been discovered, but the postcranial anatomy of *Protoceratops* has not been studied in detail. A new, mostly articulated subadult individual provides an excellent opportunity for us to comprehensively describe the anatomy of the limb skeleton, to compare to other ceratopsian dinosaurs, and to study the ontogenetic and intraspecific variation in this species. New data provided by the specimen shed light on the lifestyle of *P. andrewsi*. The young subadult individuals present an array of morphological characters intermediate between the bipedal *Psittacosaurus* and fully quadrupedal adult *P. andrewsi*. We compare these observations with a broad range of non-ceratopsid Neoceratopsia (of various locomotor adaptations) and Psittacosauridae (obligate bipeds), which gives us insight into the evolution of the skeletal characters informative for the postural change in ceratopsian dinosaurs.

## Introduction

*Protoceratops andrewsi* is one of the most common dinosaurs in the Djadokhta Formation (Upper Cretaceous; Dashzeveg et al., 2005) of the Gobi Desert, Mongolia. Numerous, sometimes complete, skeletons of these animals are known from the Bayn Dzak (=Shabarakh Usu; Brown & Schlaikjer, 1940; Jerzykiewicz & Russell, 1991), Toogreek and Tugrikin Shireh (Fastovsky et al., 1997) localities in Mongolia. The abundance of finely preserved specimens has led to many studies of functional morphology (Tereshchenko, 1994, 1996, 2008), bone microanatomy (Fostowicz-Frelik & Słowiak, 2018), behavior (Fastovsky et al., 2011; Hone et al., 2014; Hone, Wood & Knell, 2016a), ontogeny and development (Dodson, 1976; Handa, Watabe & Tsogtbaatar, 2012; Hone et al., 2014; Erickson et al., 2017), and intra-specific variability (Maiorino et al., 2015) of this dinosaur. Thus far, mainly cranial material has been examined, and the postcranial skeleton and its functional implications have been only partially studied (Senter, 2007; Maidment & Barrett, 2014). Although the postcranial skeleton of *Protoceratops andrewsi* was described by Brown & Schlaikjer (1940), most of their detailed descriptions concern bones of mature specimens and information about ontogenetic changes is limited. Moreover, they considered the sample of *Protoceratops* at their disposal (ca. 40 individuals) to be homogeneous. However, this may not be the case, because the sample from Bayn Dzak studied by Tereshchenko & Alifanov (2003) was implied to contain a hitherto unknown ceratopsian (*Bainoceratops*). Thus, our paper includes the first detailed description of an almost complete and mostly articulated limb skeleton of a new subadult specimen (ZPAL MgD-II/3) of *Protoceratops andrewsi*, which we compare with all available non-ceratopsid ceratopsians. Moreover, we give particular attention to the changes of morphology occurring during ontogeny, in order to determine any age dependent morphological trends, especially related to mode of locomotion (see similar studies on other ornithischian genera: Norman, 1980; Heinrich, Ruff & Weishampel, 1993; Dilkes, 2001; Wosik, Goodwin & Evans, 2018).

*Protoceratops* was traditionally regarded as a typical quadrupedal dinosaur (Brown & Schlaikjer, 1940; You & Dodson, 2004). Also, most of the recent, mainly morphometric, analyses classified non-ceratopsid neoceratopsians as quadrupeds (Tereshchenko, 1996; Paul & Christiansen, 2000; Chinnery, 2004a), or facultative bipeds (Senter, 2007; Maidment & Barrett, 2014). However, some species specifically were proposed as better adapted for walking on two legs (e.g., *Leptoceratops*, Maidment & Barrett, 2014; *Cerasinops*, Chinnery & Horner, 2007; or *Udanoceratops*, Chinnery, 2004a). On the other hand, the exclusively bipedal *Psittacosaurus* has been challenged as such, and facultative quadrupedality (Osborn, 1924; Sereno, 1990, 1997; You & Dodson, 2004) or ontogenetically variable gaits have been proposed (Zhao et al., 2013; Hedrick et al., 2014).

In our study, we present a thorough summary and analysis of the stance-related skeletal features in non-ceratopsid Neoceratopsia and discuss the locomotor abilities of *Protoceratops*, throughout its ontogeny. Therefore, an almost complete subadult skeleton of *Protoceratops andrewsi* allows us to consider ontogenetic variation in gait in that species.

## Materials and Methods

**Figure 1:**
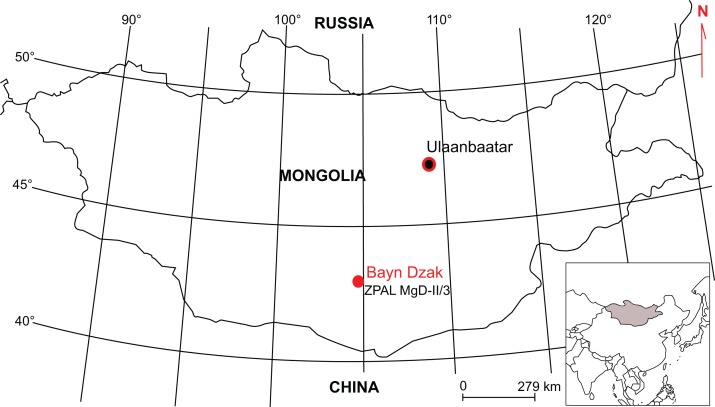
Map indicating locality of origin of ZPAL MgD-II/3 skeleton.

The specimen ZPAL MgD-II/3 was discovered in 1965 during the Polish–Mongolian expedition in Bayn Dzak (Gobi Desert, Mongolia; Fig. 1), the stratotype locality of the Djadokhta Formation, late Campanian in age (Dashzeveg et al., 2005). The bones were embedded in a matrix of fine-grained arkose sandstone of reddish-orange color and eolian origin (Gradziński, Kielan-Jaworowska & Maryańska, 1977; Loope et al., 1998; Jerzykiewicz, 2000). The slab was initially surface cleaned and displayed in the Museum of the Earth, Polish Academy of Sciences, Warsaw (for illustration, see Niedźwiedzki et al., 2012: fig 1A), but has not been studied in detail until quite recently. In 2006, all of the bones were removed from the matrix for study. The specimen is an almost complete, semi-articulated skeleton of a subadult *Protoceratops andrewsi*. The individual was lying on its right side; thus, the missing bones (which are mainly from the left side of the body) most probably were destroyed by erosion. The head was turned upside-down. The ribs are in various states of preservation; they are mostly complete but none of them is articulated (they are mostly displaced on the left side of the animal in the abdominal region). Most of the appendicular skeleton (apart from the clavicles, left femur and ilium, both pubic bones and fragments of the manus and pes) was present in the slab and partly articulated.

Apart from this specimen, we examined *Protoceratops andrewsi* individuals housed in the collections of AMNH, NHMW, PIN, and ZPAL (Table S1). The long bones were measured with a standard electronic caliper with an accuracy of 1.0 mm (see Table 1). To estimate locomotor adaptations among non-ceratopsid ceratopsians we used the tibia-to-femur length ratio, which was calculated and plotted with PAST v. 3.20 software (Hammer, Harper & Ryan, 2001). For *Psittacosaurus lujiatunensis* we added the metric data from Hedrick et al. (2014), for other data see Tables S2 and S3.

**Table 1:** Measurements of skeletal elements of *Protoceratops andrewsi* ZPAL MgD-II/3 (in mm).

Element	Measurement		
		Left	Right
Scapula	Total length	90*	129
	Dorsoventral height of proximal plate	30	30
	Dorsoventral height of distal blade	70*	100
	Dorsoventral height of glenoid fossa	15	20
	Transverse width of glenoid fossa	10	10
Coracoid	Maximum dorsoventral height	40*	?
	Maximum anteropsterior length	40*	?
	Anteroposterior length of glenoid fossa	10	?
	Transverse width of glenoid fossa	8	?
Sternal plate	Total length	40	–
Humerus	Total length	105	90*
	Maximum width of proximal end	20*	?
	Minimum shaft circumference	40	?
	Minimum shaft width	7	?
	Maximum width of distal end	25	?
	Length of deltopectoral crest	50	?
Ulna	Total length	90	75*
	Shaft width in mid-length	12	7*
	Minimum shaft circumference	30*	32
	Transverse width of distal end	13	?
	Transverse width of proximal end	24	?
Radius	Total length	75	75
	Minimum shaft width	6*	8
	Minimum shaft circumference	20	20
	Transverse width of distal end	15	15
	Transverse width of proximal end	10	15
Metacarpal II	Total length	30	–
	Transverse width of ventral articular surface	11	–
Metacarpal V	Total length	17	–
	Transverse width of ventral articular surface	9*	–
Ungual 1	Total length	14*	–
	Maximum transverse width	8*	–
Ungual 2	Total length	10*	–
	Maximum transverse width	8*	–
Ungual 3	Total length	12*	–
	Maximum transverse width	7*	–
Ilium	Total length	–	147
	Length of preacetabular process	–	55
	Length of postacetabular process	–	55
	Supracetabular height	–	30
Ischium	Total length	168*	130
	Shaft height posterior to proximal plate	128*	–
	Anteroposterior length of iliac process	10*	–
	Dorsoventral height of pubic process	10*	–
Femur	Total length	–	100*
	Maximum width of proximal end	–	30
	Mid-shaft circumference	–	60*
	Mid-shaft width	–	20*
	Dorsoventral length of fourth trochanter	–	20
Tibia	Total length	–	140*
	Mid-shaft circumference	–	60
	Mid-shaft width	–	17
	Transverse width of distal end	–	35
	Anteroposterior width of proximal end	–	34*
Fibula	Total length	–	90*
	Maximum width of proximal end	–	15*
	Maximum width of distal end	–	–
	Mid-shaft circumference	–	?
	Mid-shaft width	–	5
Metatarsal I	Total length	–	45
	Transverse width of ventral articular surface	–	12
Metatarsal II	Total length	–	65*
	Transverse width of ventral articular surface	–	–
Metatarsal III	Total length	–	51
	Transverse width of ventral articular surface	–	–
Metatarsal IV	Total length	–	57
	Transverse width of ventral articular surface	–	8
Ungual 1	Total length	–	25*
	Maximum transverse width	–	20
Ungual 4	Total length	–	30*
	Maximum transverse width	–	18*

**Note:**

Asterisk denotes approximate dimension.

To assess ontogenetic age of an animal, we followed Handa, Watabe & Tsogtbaatar (2012) when the skull was associated with the skeleton; otherwise we used Hone et al. (2014) and Hone, Farke & Wedel (2016b). We consider juveniles as specimens without any signs of maturity (AMNH 6419, PIN 3143/6, and MPC-D 100/530), and subadults as representing a mixture of features of juveniles and adults (e.g., ZPAL MgD-II/3, MgD-II/35). VS Tereshchenko (2018, unpublished data) further divided the adult stage into younger/smaller adult (e.g., PIN 3143/5, PIN 3143/7, AMNH 6470, and 6481), larger adult (e.g., AMNH 6417, 6678), and senile (“old”) individuals (e.g., AMNH 6424, 6466, PIN 3143/4). These stages can be recognized in the fusion of specific vertebra and the direction of the spinous process in the caudal vertebrae (VS Tereshchenko, 2018, unpublished data). For the sake of clarity, we generally used a simplified distinction into three main ontogenetic stages: juvenile, subadult, and adult in the present paper.

For a list of comparative material used in our study, see Table S1.

## Results

### Morphology of the pectoral girdle and forelimb

#### Scapula

**Figure 2:**
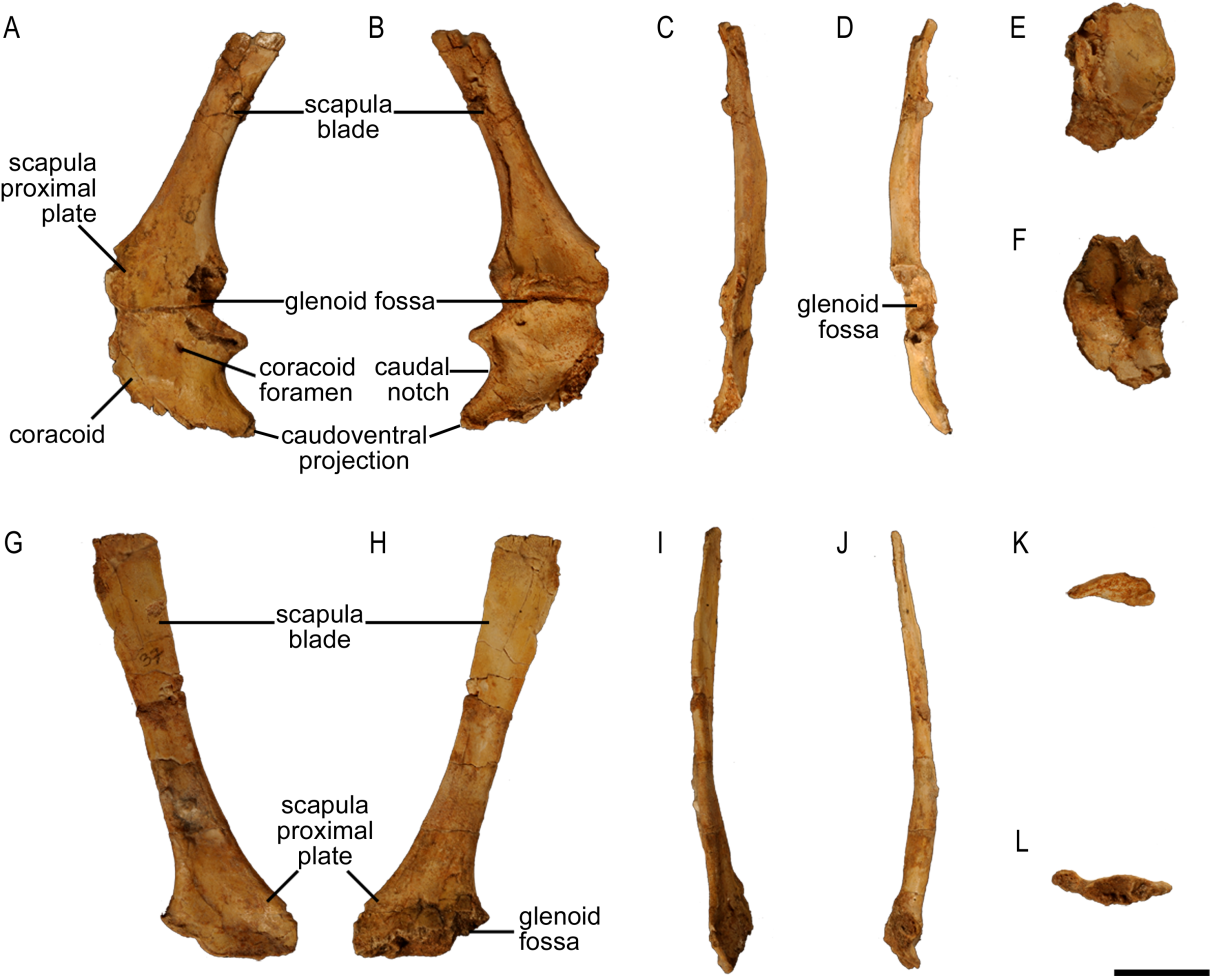
Scapula of *Protoceratops andrewsi* (ZPAL MgD-II/3, subadult). (A–D) Left scapula associated with coracoid in (A) lateral, (B) medial, (C) dorsal, and (D) ventral views. (E and F) Right coracoid in (E) lateral and (F) medial views. (G–L) Right scapula in (G) lateral, (H) medial, (I) dorsal, (J) ventral, (K) proximal, and (L) distal views. Scale bar: three cm.

**Figure 3:**
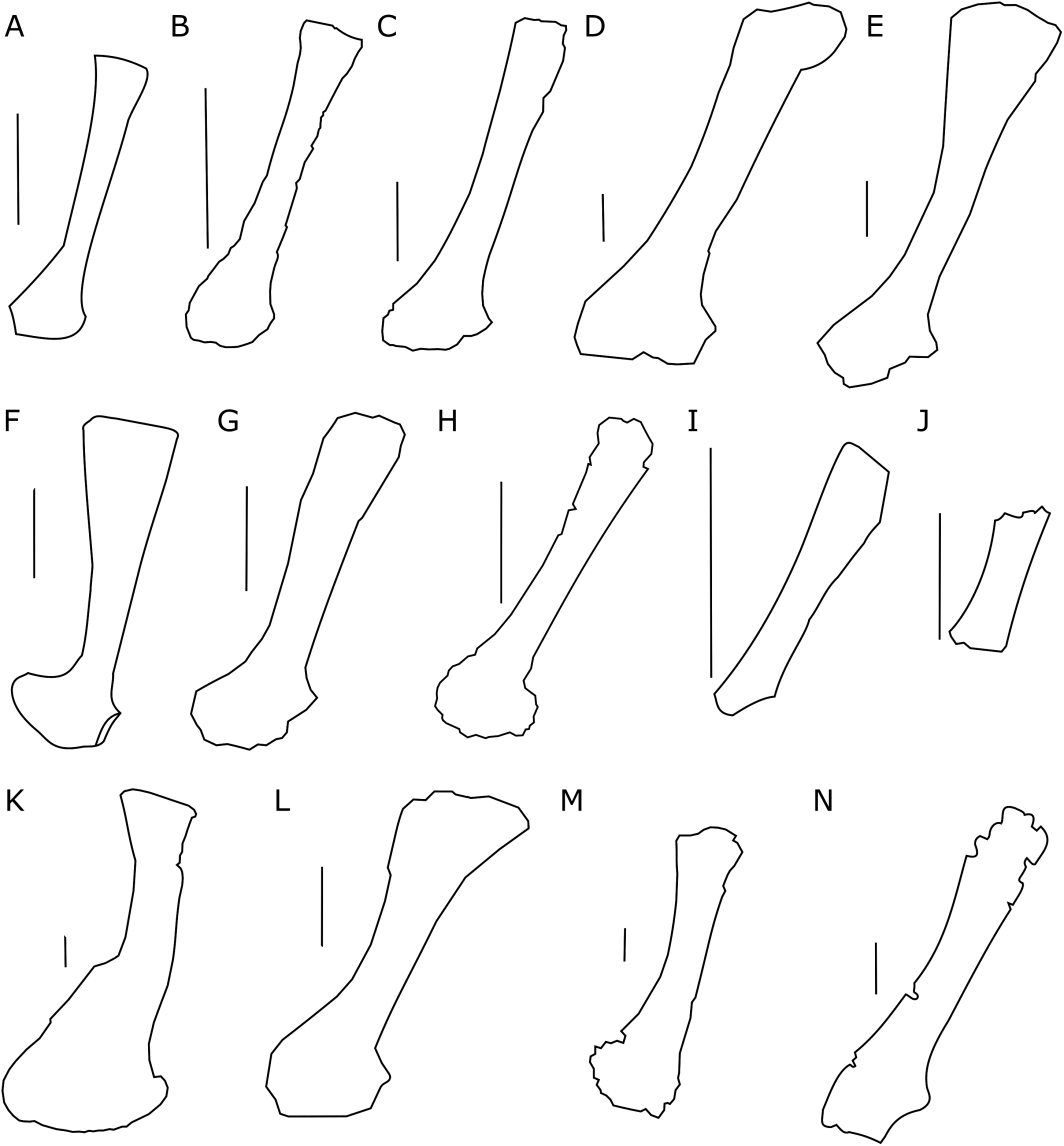
Scapula outlines. Right scapula outline in basal Ceratopsia, lateral view. (A) Hatchling of *Protoceratops andrewsi* MPCD-100/530; (B) juvenile of *P. andrewsi* AMNH 6419; (C) subadult *P. andrewsi* ZPAL MgD-II/3; (D) adult *P. andrewsi* AMNH 6418; (E) adult *P. andrewsi* AMNH 6424; (F) *Yinlong downsi* IVPP V14530; (G) *Psittacosaurus mongoliensis NHMW* 1998z0064/0001; (H) *Graciliceratops mongoliensis* ZPAL MgD-I/156; (I) *Breviceratops kozlowskii* ZPAL MgD-I/117; (J) *Archaeoceratops yujingziensis* CASG-IG-VD-0003; (K) *Leptoceratops gracilis* AMNH 5208; (L) *Auroraceratops rugosus* GSGM (07)7-04; (M) *Cerasinops hodgskissi* MOR 300; (N) *Montanoceratops cerorhynchus* MOR 542. Scale bar: one cm for (A) and three cm for (B–K). Outlined from: Fastovsky et al. (2011) (A), Han et al. (2017) (F), You, Tanoue & Dodson (2010) (J), Morschhauser (2012) (L), Chinnery & Horner (2007) (M), and Chinnery & Weishampel (1998) (N).

The scapula of *Protoceratops andrewsi* (Fig. 2) consists of a proximal plate, sub-triangular in shape, and an elongated blade (slightly shorter than three times the maximum width of the proximal plate). In adult *Protoceratops andrewsi* the scapular blade widens slightly craniocaudally near the distal end (Brown & Schlaikjer, 1940) and is more convex laterally (Figs. 3A–3E); whereas in subadult ZPAL MgD-II/3, the left scapula does not show such distal flaring (see Figs. 2G–2J for a right scapula). The blade is sub-oval in transverse section and quite thick near the caudal border, narrowing cranially. Interestingly, the scapular blade of the subadult ZPAL MgD-II/3 seems to be stouter and shows a lesser narrowing at mid-length than it is in the adult (including senile) individuals (e.g., AMNH 6417, AMNH 6424). The relatively narrow distal end of the scapular blade occurs also in juvenile *Protoceratops andrewsi* (MPCD-100/530, AMNH 6419, PIN 3143/6), *Graciliceratops* (ZPAL MgD-I/156; Fig. 3H), and *Breviceratops* (ZPAL MgD-I/117; Fig. 3I). On the other hand, a wider scapular blade is observed in smaller but mature specimens of *Protoceratops andrewsi* (PIN 3143/5, AMNH 6418, Fig. 3D), similar to the condition in *Psittacosaurus mongoliensis* (NHMW 1998z0064/0000, AMNH 6537, 6544, IVPP CV738, RV96001, V12088-2; Fig. 3G), and *Yinlong* (Han et al., 2017; Fig. 3F). The scapular blade is similarly well-developed in *Leptoceratops* (AMNH 5208; Fig. 3K)*, Auroraceratops* (Morschhauser, 2012; Fig. 3L), and *Cerasinops* (Chinnery & Horner, 2007; Fig. 3M). Furthermore, *Montanoceratops* has a flared distal end of the scapula even in juvenile specimen (Chinnery & Weishampel, 1998; Fig. 3N).

A scapular crest, extending from the caudal side of the supraglenoid ridge to the cranial part of the blade, forms a low ridge in adult *Protoceratops andrewsi* (e.g., AMNH 6418). The crest becomes more massive in old individuals (e.g., AMNH 5424), but the structure is barely visible in ZPAL MgD-II/3 (Fig. 2G), which is a comparatively immature specimen.

The scapula articulates with the coracoid along a straight suture (Fig. 2A), and the long axis of the scapular blade forms an angle of about 70° with the scapulocoracoid suture. The blade is almost vertical in *Leptoceratops* (AMNH 5208) and *Cerasinops* (Chinnery & Horner, 2007), but slightly oblique in senile *Protoceratops* (e.g., AMNH 6424) and *Montanoceratops* (Chinnery & Weishampel, 1998). It is more oblique in *Psittacosaurus* (e.g., NHMW 1998z0064/0001), and even more so in *Auroraceratops* (Morschhauser, 2012) and *Graciliceratops* (ZPAL MgD-I/158).

**Figure 4:**
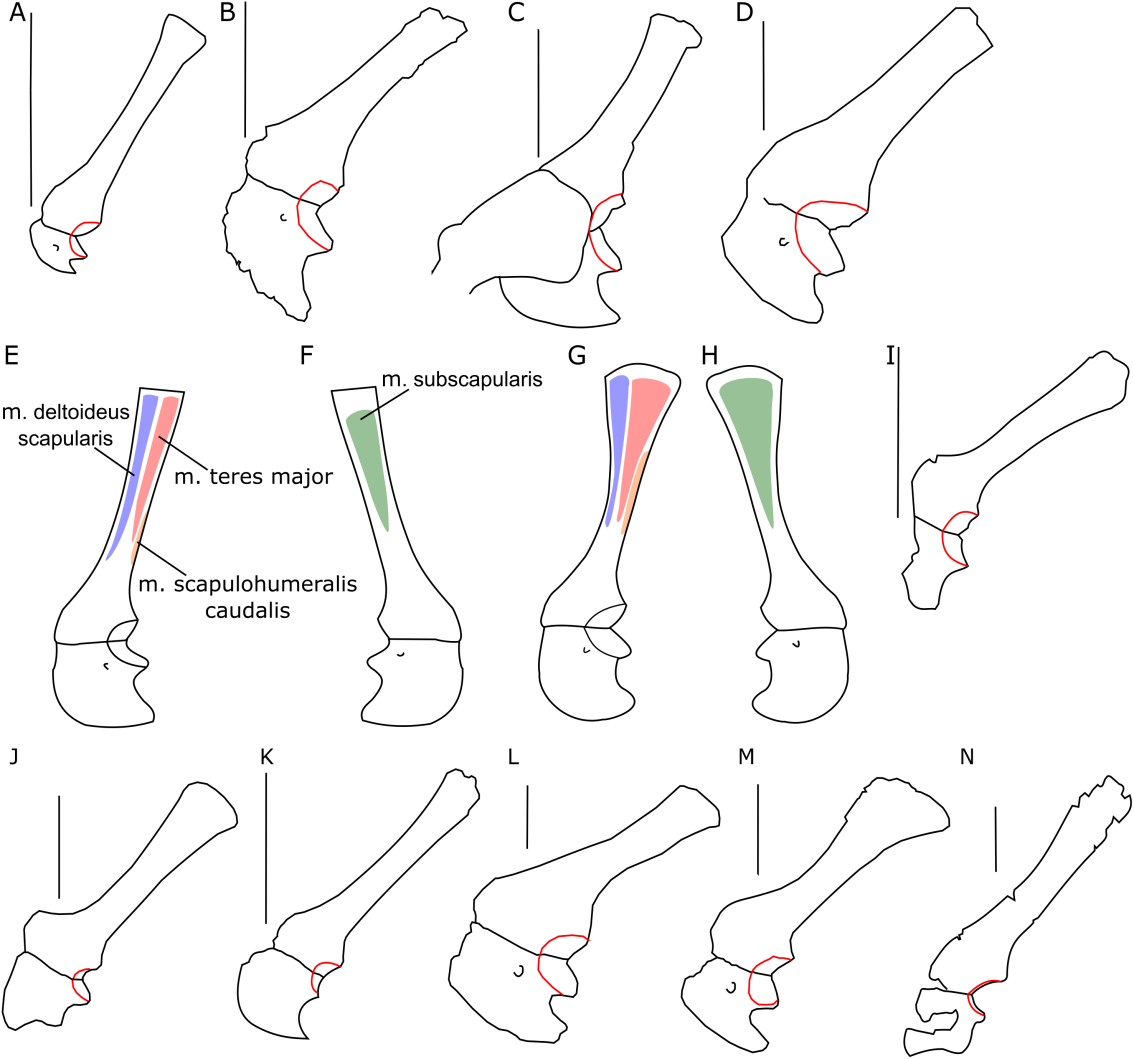
Scapulocoracoid outline. Left scapulo-coracoid outline in basal Ceratopsia, lateral view (A–D, I–N) and muscle attachment areas on scapula-coracoid of *Protoceratops andrewsi* (E–H). (A) Juvenile of *P. andrewsi* AMNH 6419; (B) subadult *P. andrewsi* ZPAL MgD-II/3; (C) adult *P. andrewsi* AMNH 6418; (D) adult *P. andrewsi* AMNH 6471; (E and F) muscle attachment areas on ZPAL MgD-II/3 scapulo-coracoid in (E) lateral and (F) medial view; (G and H) muscle attachment areas on adult *P. andrewsi* (AMNH 6424, 6418, 6471) scapulo-coracoid in (G) lateral and (H) medial views; (I) *Psittacosaurus mongoliensis* NHMW 1998z0064/0001; (J) *Xuanhuaceratops niei* IVPP 12722; (K) *Graciliceratops mongoliensis* ZPAL MgD-I/156; (L) *Leptoceratops gracilis* AMNH 5205; (M) *Auroraceratops rugosus* GSGM (07)7-04; (N) *Montanoceratops cerorhynchus* MOR 542. Margin of glenoid surface indicated by red line. Scale bar: five cm. Outlined from: Zhao et al. (2006) (J), Morschhauser (2012) (M), and Chinnery & Weishampel (1998) (N).

The glenoid articular surface is semicircular, deeply concave, and faces caudolaterally. This oblique orientation is an indication of the caudolateral, instead of directly caudal, position of the humerus. The widening of the glenoid cavity varies among neoceratopsians (Fig. 4). It is very small in *Psittacosaurus mongoliensis* (e.g., AMNH 6535, 6534, NHMW 1998z0064/0001; Fig. 4I), *Graciliceratops* (ZPAL MgD-I/156; Fig. 4K), juvenile *Protoceratops andrewsi* (AMNH 6419, PIN 3143/6; Fig. 4A), and juvenile *Montanoceratops* (Chinnery & Weishampel, 1998; Fig. 4N). The glenoid expands more laterally in small subadult *Protoceratops andrewsi* (ZPAL MgD-II/3; MgD-II/35; Fig. 4B) and a lateral extension of the glenoid is similar to that in *Xuanhuaceratops* (Zhao et al., 2006; Fig. 4J). Adult *Protoceratops andrewsi* (young adults: AMNH 6418, AMNH 6471, PIN 3143/5, PIN 3143/7; large adults: AMNH 6417; and senile individuals: AMNH 6424, PIN 3143/4) have very different ranges of the lateral extension of the glenoid surface despite their similar ontogenetic status. In *Leptoceratops* (AMNH 5205; Fig. 4L) and *Auroraceratops* (Morschhauser, 2012; Fig. 4M), the glenoid is more laterally expanded than in AMNH 6418, but less than in AMNH 6471. The expansion of the glenoid appears to have a complex distribution among taxa (Fig. 4), and the lack of the expanded glenoid was most probably compensated for by cartilage, playing an important role in the structure of the joint. Thus, the glenoid position may not be indicative of gait through ontogeny in *Protoceratops*.

#### Coracoid

Both bones are present. The left coracoid is well preserved in ZPAL MgD-II/3, but its cranioventral margin is damaged (Figs. 2A, 2B, 2E and 2F). Its external (lateral) surface is slightly concave. The caudal margin bears the glenoid fossa. In ZPAL MgD-II/3, the coracoid portion of the glenoid cavity is overall larger than that of the scapular plate, similar to the condition found in adult *Protoceratops andrewsi* (Figs. 4C and 4D). The caudal margin of the coracoid is concave, forming a caudal notch with the caudoventral corner drawn out into a tapering process (Figs. 2A and 2B). In medial view, a small groove, continuous with the coracoid foramen, is visible at the caudal part of the bone. The coracoid foramen is elliptical; its long axis oriented craniocaudally. The coracoid bone is thin cranially, but thickens caudally.

The outline of the coracoid is similar in all Ceratopsia (Fig. 4). The caudoventral projection occurs in non-ceratopsid neoceratopsians. The caudal notch is shallow in *Psittacosaurus mongoliensis* (NHMW 1998z0064/0001), *Xuanhuaceratops* (Zhao et al., 2006), *Graciliceratops* (ZPAL MgD-I/156), and young *Montanoceratops* (Chinnery & Weishampel, 1998). By contrast, in *Leptoceratops* (AMNH 5205), *Auroraceratops* (Morschhauser, 2012) and *Protoceratops* (e.g., AMNH 6471), the caudal notch is deep and narrow (Fig. 4). In ZPAL MgD-II/3, the caudal notch is wider dorsoventrally than in adult *Protoceratops andrewsi*, although it may be partly caused by a mediolateral compression due to the fossilization process, because the notch is deep and narrow in most specimens of *Protoceratops andrewsi* (e.g., in juvenile AMNH 6419, and small adults AMNH 6418 and 6471). The cranioventral margin of the coracoid is rounded in *Protoceratops andrewsi* (e.g., AMNH 6418, and PIN 3143/4) and *Graciliceratops* (ZPAL MgD-I/156). A similar, but less convex cranioventral margin of the coracoid occurs in *Leptoceratops* (AMNH 5205), *Auroraceratops* (Morschhauser, 2012), and *Protoceratops andrewsi* (AMNH 6471). Also, a concavity occurs at the ventral margin of the coracoid in *Psittacosaurus mongoliensis* (e.g., NHMW 1998z0064/0001), while its cranial margin is quite straight (Fig. 4).

#### Sternal plates

**Figure 5:**
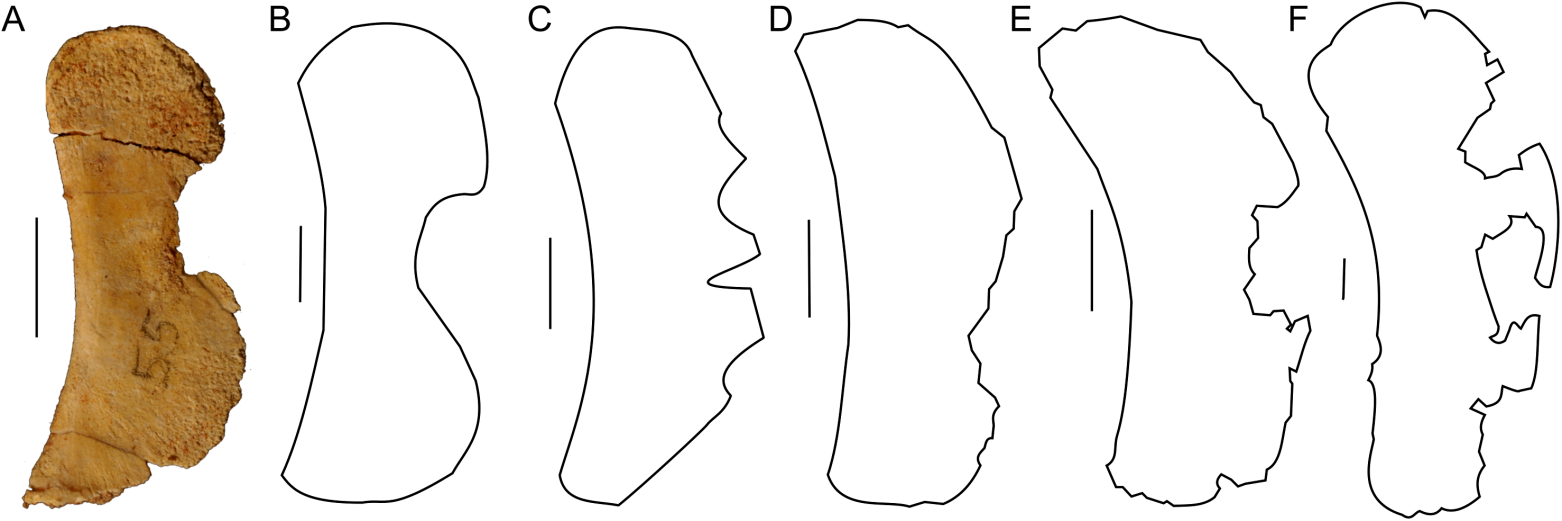
Sternal plate morphology. Photograph of ZPAL MgD-II/3 and outline of basal Ceratopsia sternal plates in ventral view. (A) Subadult *Protoceratops andrewsi* ZPAL MgD-II/3; (B) adult *Protoceratops andrewsi* AMNH 6408; (C) *Psittacosaurus sibiricus* PM TGU 16/1-51; (D) *Leptoceratops gracilis* NMC 8889; (E) *Auroraceratops rugosus* GSGM (07)-24; (F) *Montanoceratops cerorhynchus* MOR 542. Scale bar: one cm. Outlined from: Averianov et al. (2006) (C), Morschhauser & Lamanna (2013) (D and E), and Chinnery & Weishampel (1998) (F).

Only a left sternal plate is preserved in ZPAL MgD-II/3 (Fig. 5A). The bone is almost complete and does not differ in shape from that of adult *Protoceratops andrewsi* (AMNH 6408; Brown & Schlaikjer, 1940; Morschhauser & Lamanna, 2013). Nonetheless, it is very thin compared to the condition in older individuals (e.g., AMNH 6408).

The lateral margin of the plate bears a wide and distinct notch. The caudal margin is more pointed than the cranial one, whereas the cranial and caudal parts of the medial margin are convex and wide. The sternum of *Protoceratops andrewsi* differs from that of other non-ceratopsid ceratopsians in having a concavity on the medial margin (Fig. 5).

#### Humerus

**Figure 6:**
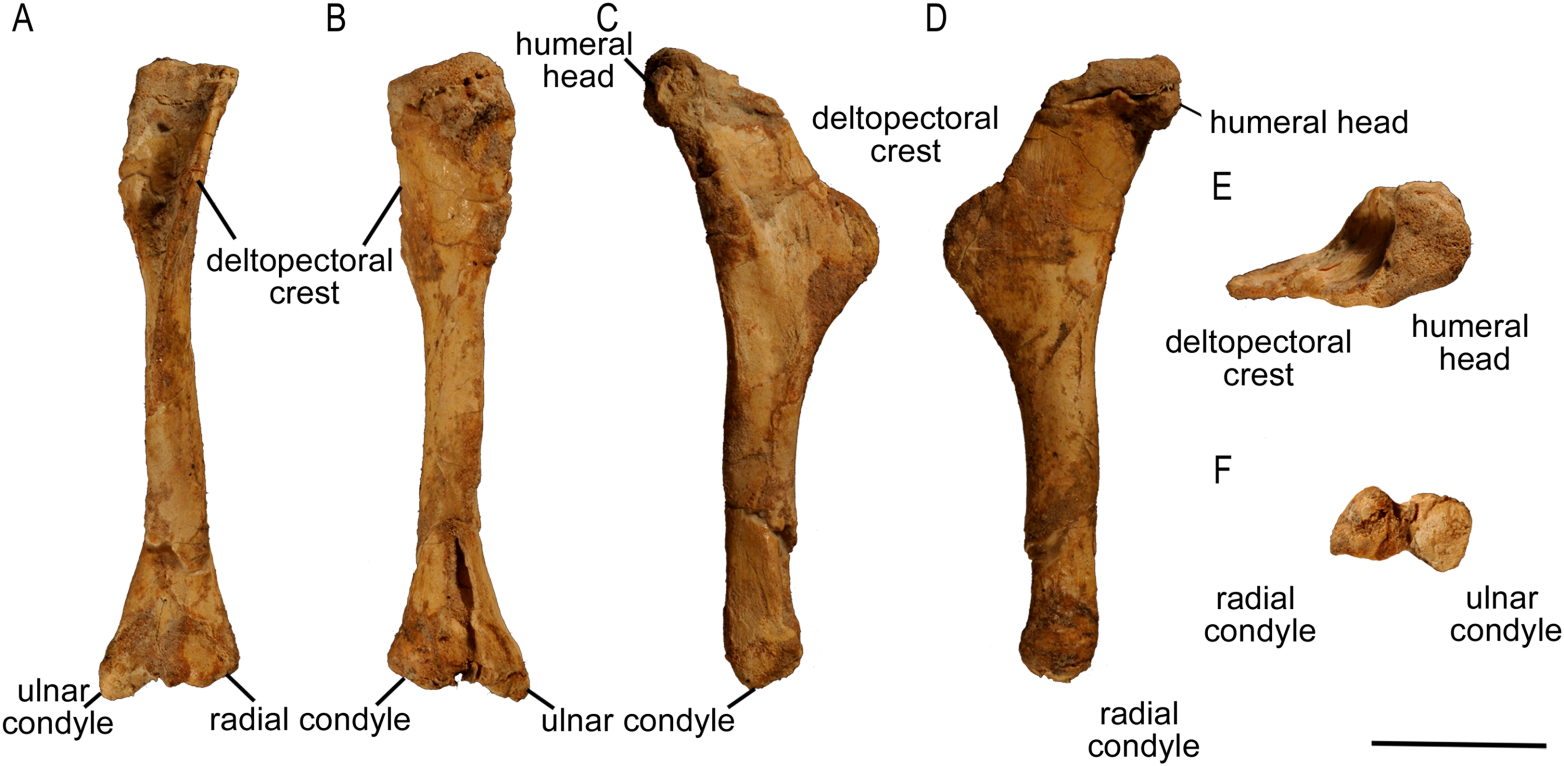
Left humerus of *Protoceratops andrewsi* (ZPAL MgD-II/3) in (A) anterior, (B) posterior, (C) medial, (D) lateral, (E) dorsal, and (F) ventral views.

The left humerus is complete and well preserved in ZPAL MgD-II/3 (Fig. 6), whereas the right element is partially damaged (the proximal extremity is broken) and distorted by compaction and erosion. The left bone displays slight torsion; the angle between the planes of both articular surfaces (at the proximal and distal ends) is about 20°. The shaft of the humerus is slender, but widens transversely at both ends. It is suboval in cross-section distally. The proximal part is inflected caudally and capped by a proximally convex humeral head protruding caudally.

**Figure 7:**
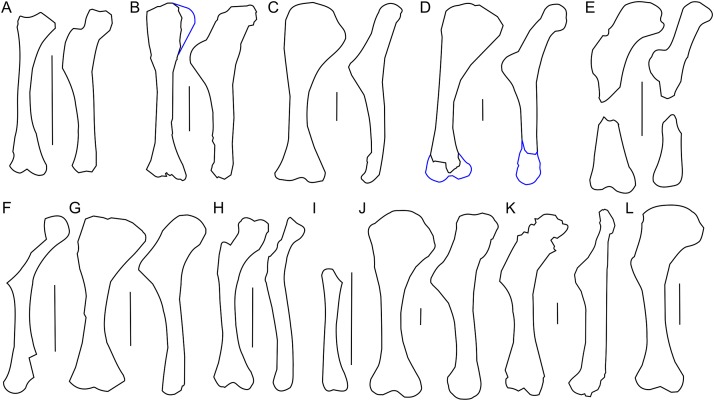
Humerus morphology. Left humerus outline in basal Ceratopsia, cranial and lateral views. (A) Juvenile *Protoceratops andrewsi* AMNH 6419; (B) subadult *P. andrewsi* ZPAL MgD-II/3; (C) adult *P. andrewsi* AMNH 6418; (D) adult *P. andrewsi* AMNH 6471; (E) *Yinlong downsi* IVPP V18679; (F) *Xuanhuaceratops niei* IVPP 12722; (G) *Psittacosaurus mongoliensis* NHMW 1998z0064/0001; (H) *Graciliceratops mongoliensis* ZPAL MgD-I/156; (I) *Breviceratops kozlowskii* ZPAL MgD-I/117; (J) *Leptoceratops gracilis* AMNH 5205; (K) *Cerasinops hodgskissi* MOR 300; (L) *Auroraceratops rugosus* GSGM (07)9-60. Missing elements are reconstructed with blue line. Scale bar: three cm. Outlined from: Han et al. (2017) (E), Zhao et al. (2006) (F), Chinnery & Horner (2007) (K), and Morschhauser (2012) (L).

The humerus of subadult *Protoceratops andrewsi* ZPAL MgD-II/3 mostly differs from those of adult specimens in the morphology of its proximal extremity (Figs. 7A–7D). Similar to the condition in juvenile *Protoceratops andrewsi* (AMNH 6419), the humerus of ZPAL MgD-II/3 has a less expanded proximal end in cranial view than the larger specimens, but it is wider craniolaterally. The proximal end of the humerus is also wide mediolaterally in *Psittacosaurus mongoliensis* (NHMW 1998z0064/0001; Fig. 7G), *Leptoceratops* (AMNH 5205; Fig. 7J) and *Auroraceratops* (Morschhauser, 2012; Fig. 7L), but is slender in *Graciliceratops* (ZPAL MgD-I/156; Fig. 7H), *Yinlong* (Han et al., 2017; Fig. 7E) and *Cerasinops* (Chinnery & Horner, 2007; Fig. 7K).

The proximal articular facet of ZPAL MgD-II/3 is transversely enlarged and subtriangular, with the base slightly concave and directed cranially, whereas the apex is directed caudally. Furthermore, the head of the humerus is inclined medially in all non-ceratopsid Ceratopsia regardless of the ontogenetic stage.

In ZPAL MgD-II/3 an overall thin deltopectoral crest projects craniolaterally (at ca. 30°) from the shaft, forms the transverse expansion of the 1/3 of the proximal part of the bone, and merges into the cranial surface of the shaft. The cranial surface of the proximal end of the humerus in ZPAL MgD-II/3 is concave, forming an elongated fossa for the attachment of the m. coracobrachialis brevis (Maidment & Barrett, 2011). The fossa is medially constricted by a low ridge, and additionally underlined by the deltopectoral crest. In adult *Protoceratops*, the deltopectoral crest is similarly located (e.g., in small adult NHMW 1015/0404/0001 and senile individual AMNH 6424), although it is much thicker, especially at its rounded margin, and the concavity of the proximal end of the humerus is shallower and wider mediolaterally. In juvenile *Protoceratops andrewsi* (AMNH 6419; Fig. 7A) the deltopectoral crest is elongate dorsoventrally. During ontogeny the proximal end of the humerus extends cranially and the deltopectoral crest forms a rounded cranial extension (see, e.g., AMNH 6424; Fig. 7D). Similar morphology can be observed in *Yinlong* (Han et al., 2017; Fig. 7E), *Cerasinops* (Chinnery & Horner, 2007; Fig. 7K), and *Leptoceratops* (AMNH 5205; Fig. 7J), whereas the deltopectoral crest is pointed in *Psittacosaurus mongoliensis* (e.g., AMNH 6537, NHMW 1998z0064/0001; Fig. 7H).

The humerus of *Cerasinops* is unique among the Ceratopsia due to its slenderness. The proximal end is nearly as wide as the distal one, but strongly inclined to the medial side, although both ends are relatively poorly preserved (Chinnery & Horner, 2007). Moreover, the deltopectoral crest is less cranially prominent in *Cerasinops* than in any other genus mentioned here. In contrast, the humerus of *Leptoceratops* (AMNH 5205) is robust and the apex of the deltopectoral crest is located more distally than in *Cerasinops* (Chinnery & Horner, 2007) and *Auroraceratops* (Morschhauser, 2012). *Leptoceratops*, *Cerasinops*, and *Auroraceratops* have broad and robust humeral shafts in contrast to ZPAL MgD-II/3 and other *Protoceratops andrewsi* specimens (AMNH 6418, 6424), as well as *Psittacosaurus mongoliensis* (NHMW 1015/0404/0001) and *Graciliceratops* (ZPAL MgD-I/156), the last displaying the most delicate humerus. In lateral view, all compared ceratopsians, except for *Cerasinops*, have arched humeri.

The distal extremity of the humerus is expanded transversely (Fig. 6). The distal condyles in ZPAL MgD-II/3 project slightly cranially and are divided by a deep midline groove that extends onto the distal surface. The condyles are equal in size, but the ulnar condyle is slightly longer distally than the radial one. In a young adult *Protoceratops* (AMNH 6418), which is larger than the subadult ZPAL MgD-II/3, the ulnar condyle is wider transversely than the radial one. The epicondyles are not developed, and there are no epicondylar foramina. Corresponding to the extent of the joint capsule of the ulnar articulation, deep and elongate subtriangular concavities are situated directly above the distal articular end on both cranial and caudal surfaces. Both structures are distinctly bordered by sharp ridges, which are more prominent in the case of the caudal concavity. The articular surfaces of the ulnar and radial condyles face mostly distally. The articular surface of the ulnar condyle, which protrudes strongly distally, is turned toward the radial condyle. In the case of the radial condyle the articular surface extends onto the cranial face of the humerus.

In all non-ceratopsid neoceratopsian genera considered herein, the ulnar condyle is more prominent than the radial one, and the olecranon fossa is shallow (note that the state of the distal condyles in *Protoceratops andrewsi* AMNH 6418 is an artifact due to craniocaudal compaction). All genera have the proximal extremity transversely wider than the distal one with an exception of *Cerasinops*, immature *Protoceratops andrewsi* (juvenile AMNH 6419 and subadult ZPAL MgD-II/3, ZPAL MgD-II/35), and *Graciliceratops* (ZPAL MgD-I/156). In adult *Protoceratops* (e.g., AMNH 6424) the proximal expansion is transversely broader than in subadult ZPAL MgD-II/3 or ZPAL MgD-II/35, and the humeral shaft and distal condyles are more robust, although in medial or lateral view ZPAL MgD-II/3 seems much wider.

#### Ulna

**Figure 8:**
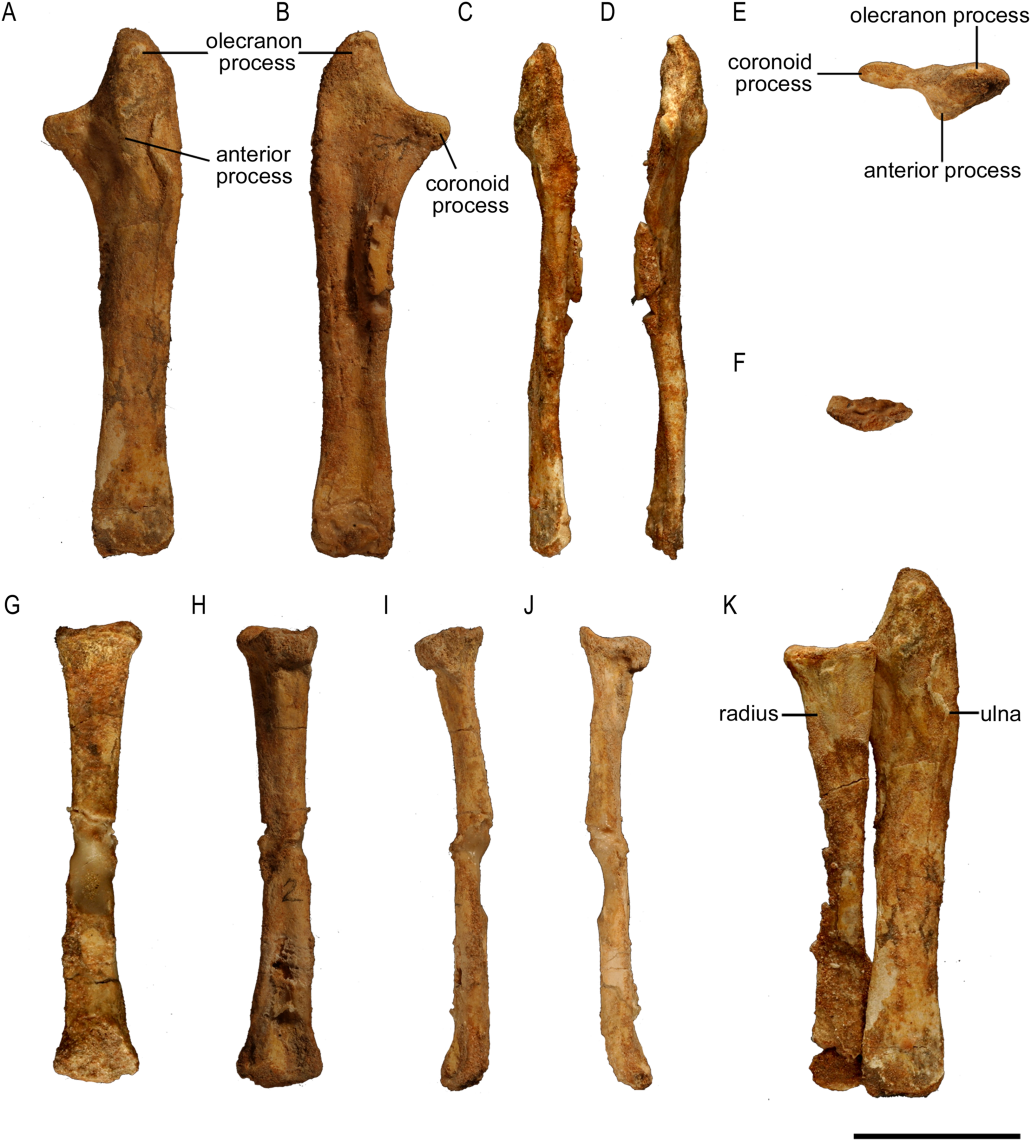
Ulna and radius of *Protoceratops andrewsi* (ZPAL MgD-II/3). A–F, Left ulna in (A) craniolateral, (B) caudomedial, (C) caudolateral, (D) craniomedial, (E) proximal, and (F) caudal views; G–J, Right radius in (G) craniolateral, (H) caudomedial, (I) caudolateral, (J) craniomedial views. K, Left forearm in craniolateral view. Scale bar: three cm.

**Figure 9:**
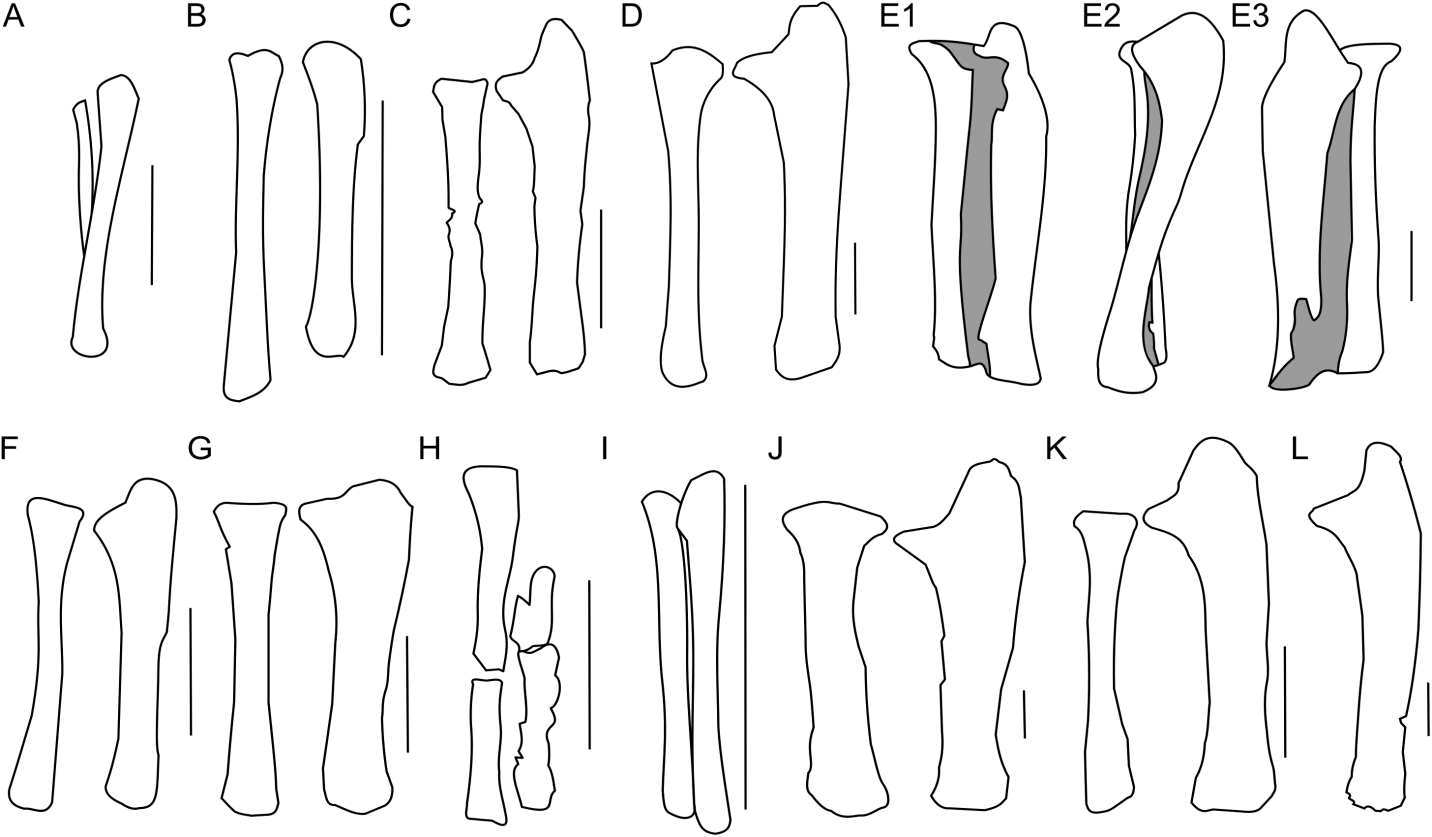
Ulna and radius morphology. Left ulna and radius outlines in basal Ceratopsia. (A) Hatchling of *Protoceratops andrewsi* MPCD-100/530 in lateral view; (B) juvenile of *P. andrewsi* AMNH 6419 in craniolateral view; (C) subadult *P. andrewsi* ZPAL MgD-II/3 in craniolateral view; (D) adult *P. andrewsi* AMNH 6424 in craniolateral view; (E) adult *P. andrewsi* AMNH 6418 in (E1) craniolateral, (E2) lateral, and (E3) caudomedial view; (F) *Yinlong downsi* IVPP V14530; (G) *Psittacosaurus mongoliensis* NHMW 1998z0064/0001 in craniolateral view; (H) *Graciliceratops mongoliensis* ZPAL MgD-I/156 in craniolateral view; (I) *Breviceratops kozlowskii* ZPAL MgD-I/117 in craniolateral view; (J) *Leptoceratops gracilis* AMNH 5205 in craniolateral view; (K) *Auroraceratops rugosus*, ulna GSGM (07)9-60 and radius GSGM (09)05 in craniolateral view; (L) *Cerasinops hodgskissi* MOR 300 in craniolateral view. Scale bar: one cm for (A) and three cm for (B–K). Outlined from: Fastovsky et al. (2011) (A), Han et al. (2017) (F), Morschhauser (2012) (K), and Chinnery & Horner (2007) (L).

The left ulna of ZPAL MgD-II/3 is complete and well preserved, but the proximal one-third of the bone is bent cranially (Fig. 8). The right ulna is poorly preserved, being crushed and lacking the proximal and distal ends. The ulna of ZPAL MgD-II/3 is very similar in shape to that of an adult *Protoceratops andrewsi* (Figs. 9D and 9E). The only difference is in the caudal margin of the ulna, which is straighter in adults, and the form of the coronoid process, which becomes more prominent (Brown & Schlaikjer, 1940).

The ulnar shaft is bilaterally flattened (due to mediolateral compression), but the proximal half is slightly convex laterally and concave medially. The cross-section of the ulna is triangular in the proximal part, but oval distally in ZPAL MgD-II/3 and all non-ceratopsid ceratopsians (You & Dodson, 2004). The caudal margin of the ulna in *Psittacosaurus mongoliensis* is sinusoidal, forming a concavity behind the expanded distal end. This feature is absent in senile *Protoceratops* (AMNH 6424) and *Yinlong* (Han et al., 2017), slightly marked in subadult (ZPAL MgD-II/3) and young adult (AMNH 6418) *Protoceratops*, and *Auroraceratops* (Morschhauser, 2012), and well developed in *Leptoceratops* (AMNH 5205) and *Cerasinops* (Chinnery & Horner, 2007).

In ZPAL MgD-II/3 the olecranon process protrudes almost 15 mm beyond the proximal end of the radius. It is triangular and flattened medially, and forms a heavy tuberosity for the insertion of the m. triceps brachii on the lateral surface. Caudal to the tuberosity, a sinuous ridge extends distally, which probably served the same function. The ridge, whose distinctness has been exaggerated by crushing of the shaft, fades out at one-third of the ulnar length. The angle between the olecranon and coronoid processes within the sigmoid notch (sensu Romer, 1970) is about 100°. The proximal articular surface is narrow, but at the coronoid process it seems to extend onto the medial side of bone. The morphology of the proximal part of the ulna in the youngest known specimens of *Protoceratops andrewsi* is not known adequately because of poor preservation; nonetheless, a juvenile specimen MPC-D 100/530 has a visible olecranon process (Fastovsky et al., 2011).

The ulna of *Psittacosaurus mongoliensis* differs from that of *Protoceratops andrewsi* in having a shallower articulation surface for the humerus, shorter olecranon process, and lower coronoid process (Fig. 9G). A short olecranon also occurs in *Breviceratops* (ZPAL MgD-I/117; Fig. 9I) and *Yinlong* (Han et al., 2017; Fig. 9F). The olecranon process of *Auroraceratops* (Morschhauser, 2012; Fig. 9K) is wider transversely than that of ZPAL MgD-II/3, but similar to that of an adult *Protoceratops andrewsi* (AMNH 6424; Fig. 9D). *Leptoceratops* (AMNH 5205; Fig. 9J) has a longer olecranon, similar to *Auroraceratops*. The olecranon of *Cerasinops* is high, but narrow (Chinnery & Horner, 2007; Fig. 9L), similar to that of ZPAL MgD-II/3 (Fig. 9C). The coronoid is strongly developed in subadult (ZPAL MgD-II/3), small adult (AMNH 6418) and senile (AMNH 6424) *Protoceratops andrewsi*, as well as in *Psittacosaurus* (NHMW 1998z0064/0001), *Leptoceratops* (AMNH 5205), *Auroraceratops* (Morschhauser, 2012), and *Cerasinops* (Chinnery & Horner, 2007). On the other hand, the coronoid is less developed in *Yinlong* (Han et al., 2017), juvenile *Protoceratops* (AMNH 6419), and *Breviceratops* (ZPAL MgD-I/117).

The distal portion of the shaft is mediolaterally flattened, but its mediocaudal surface is slightly convex. In the laterocaudal or mediocranial view the shaft is slightly concave, which is apparent in the proximal half of the bone, probably partly as a result of the crushing of the shaft. The distal end is transversely expanded and the distal articular surface is narrow craniocaudally.

The distal end of the ulna is narrow mediolaterally in juvenile and subadult *Protoceratops andrewsi* (MPC-D 100/530, ZPAL MgD-II/3), and in *Cerasinops* (Chinnery & Horner, 2007), in which it may be a matter of preservation. It is overall more expanded (in all directions) in adult *Protoceratops andrewsi* (AMNH 6424), *Psittacosaurus mongoliensis* (NHMW 1998z0064/0001), *Leptoceratops* (AMNH 5205), and *Auroraceratops* (Morschhauser, 2012). The distal end of the ulna is convex in *Psittacosaurus mongoliensis*, *Yinlong* (Han et al., 2017), and *Breviceratops* (ZPAL MgD-I/117), but straight in other ceratopsians considered (Fig. 9).

#### Radius

The left radius of ZPAL MgD-II/3 is complete, but the shaft is crushed; on the other hand, the right radius seems to be better preserved, but has been damaged in the mid-shaft region, and some bone is missing (Fig. 8). The shaft is bilaterally flat, the lateral margin is straight, and the medial margin is concave. The proximal end of the radius bears an elongate oval facet. The narrow articular facet is slightly bent to the medial side, corresponding to the concavity of the medial surface of the shaft. If it is not a result of distortion, the convex, distal, articular end of the radius faces medially (Figs. 8G–8J). The radius of juvenile (AMNH 6419) and subadult (ZPAL MgD-II/3) is slender, similar to *Yinlong* (Han et al., 2017; Fig. 9F), *Breviceratops* (ZPAL MgD-I/117; Fig. 9I), and *Graciliceratops* (ZPAL MgD-I/156; Fig. 9H). The radii of *Psittacosaurus mongoliensis* (Sereno, 1987), *Protoceratops* at all ontogenetic stages (AMNH 6419, AMNH 6424; Figs. 9D and 9E), and *Auroraceratops* (Morschhauser, 2012; Fig. 9K) are also slender in cranial view, but overall more massive. In *Leptoceratops* (AMNH 5205; Fig. 9J) the radius is even more massive, being only slightly less robust than the ulna. In all mentioned species the proximal end of the radius is expanded in all views and bears a concave articular surface. The proximal part of the shaft is triangular in cross-section in *Protoceratops andrewsi* (e.g., subadult ZPAL MgD-II/3, small adult AMNH 6418) and *Auroraceratops* (Morschhauser, 2012), but subcircular in *Leptoceratops* (AMNH 5205) and *Psittacosaurus mongoliensis* (NHMW 1998z0064/0001). The distal extremity is subcircular in *Psittacosaurus mongoliensis* (NHMW 1998z0064/0001) and *Leptoceratops* (AMNH 5205), some specimens of *Protoceratops andrewsi* (subadult ZPAL MgD-II/3, small adult AMNH 6418) and in *Auroraceratops* (Morschhauser, 2012), although in other specimens it can be rather tear-shaped. In all species the distal end is craniocaudally flattened. However, this flattening is expressed most strongly in *Protoceratops*, *Psittacosaurus mongoliensis*, and *Auroraceratops* (Morschhauser, 2012), whereas in *Leptoceratops* it seems expressed least strongly (Fig. 9).

#### Manus

**Figure 10:**
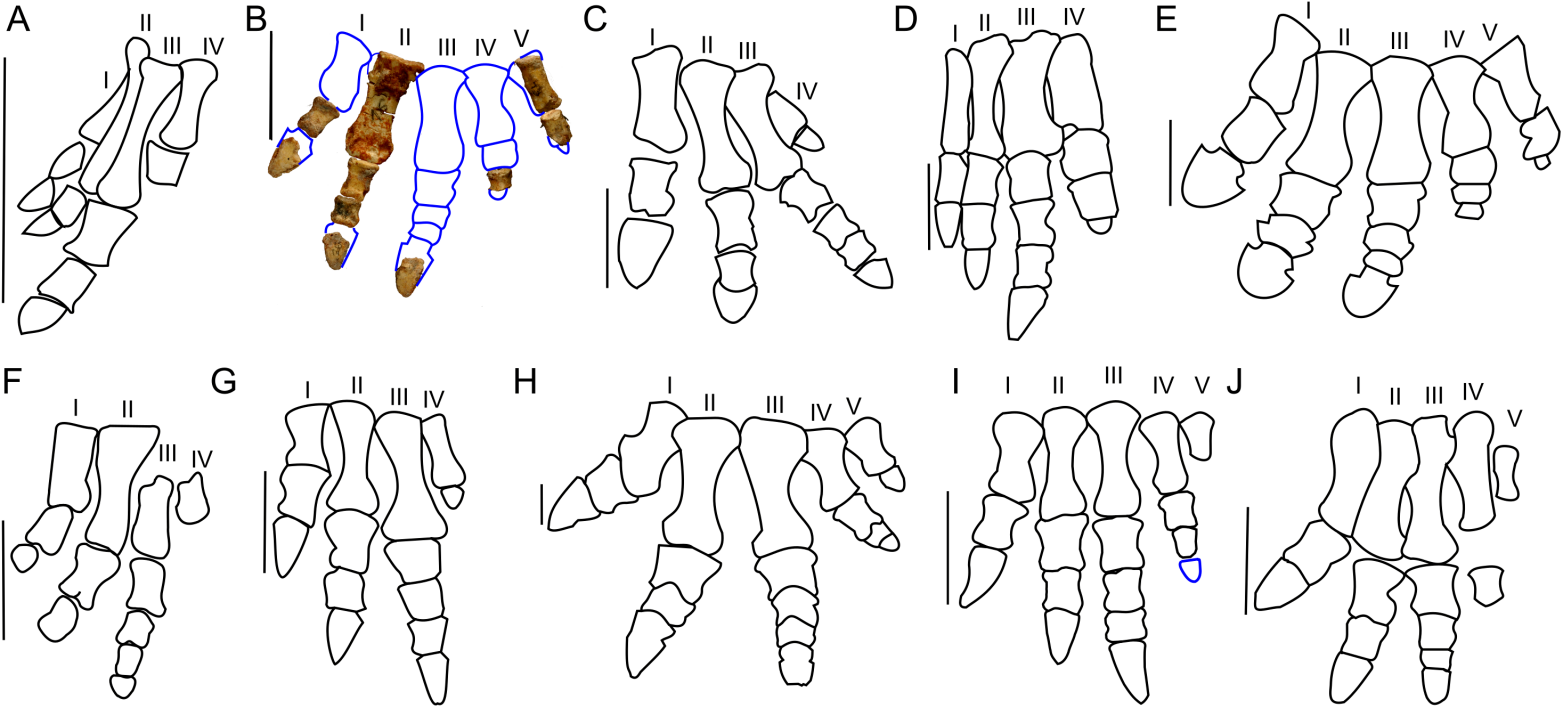
Manus morphology. Manus of basal Ceratopsia. (A) hatchling of *Protoceratops andrewsi* MPCD-100/530; (B) subadult *P. andrewsi* ZPAL MgD-II/3; (C) adult *P. andrewsi* AMNH 6418; (D) adult *P. andrewsi* NHMW 2015/0404/0001; (E) adult *P. andrewsi* AMNH 5351; (F) *Yinlong downsi* IVPP V14530; (G) *Psittacosaurus mongoliensis* AMNH 6254; (H) *Leptoceratops gracilis* AMNH 5205; (I) *Auroraceratops rugosus*, GSGM (09)05, GJ<07>07-04, GJ<08>-6; (J) *Montanoceratops cerorhynchus* MOR 542. Scale bar: one cm for (A) and three cm for (B–J). Outlined from: Fastovsky et al. (2011) (A), Brown & Schlaikjer (1940) (E), Han et al. (2017) (F), Morschhauser (2012) (I), and Chinnery & Weishampel (1998) (J).

In ZPAL MgD-II/3 the carpal elements are not preserved, and only the incomplete left manus was found (Fig. 10B). The only preserved metacarpals, namely the second and fifth, are similar in overall shape to those of adult *Protoceratops andrewsi*. In the manus of *Protoceratops*, the metacarpal II is the longest, metacarpal III is marginally shorter, metacarpal I is even shorter and nearly equal in length to metacarpal IV, and the metacarpal V is shortest of all (Figs. 10A–10E).

Metacarpal II of ZPAL MgD-II/3 bears a convex proximal articular surface. In cranial view the proximal and distal ends are widened. The lateral condyle is more transversely prominent than the medial condyle. The second metacarpal becomes more flattened distally. Metacarpal V is poorly preserved, but is much shorter and more slender than metacarpal II. The proportions of the metacarpals in *Protoceratops* (AMNH 6418, NHMW 2015/0404.001) are similar to those in *Psittacosaurus mongoliensis* (AMNH 6254, 6260; Fig. 10G). In other non-ceratopsid Neoceratopsia, including *Auroraceratops* (Morschhauser, 2012; Fig. 10I), *Leptoceratops* (AMNH 5205; Fig. 10H), and *Montanoceratops* (Chinnery & Weishampel, 1998; Fig. 10J) metacarpal III is longer than metacarpal II; the difference in length between these bones is most pronounced in *Auroraceratops* (Fig. 10).

*Protoceratops andrewsi* has the phalangeal formula 2-3-4-3-2, typical of Ceratopsia, and the non-ungual phalanges grow shorter from proximal to distal (You & Dodson, 2004). Only six phalanges of the left manus ZPAL MgD-II/3 are preserved and they were assigned positions based on the manus of small adult *Protoceratops* (NHMW 2015/0404/001 and AMNH 6418). Two phalanges of the digit I are preserved; the distal part of the pointed ungual resembles those of *Yinlong* (Xu et al., 2006), *Psittacosaurus mongoliensis* (Sereno, 1987), and *Auroraceratops* (Morschhauser, 2012) in bearing neurovascular grooves laterally and medially (Fig. 10). Digit II is nearly complete, in that two non-ungual phalanges and the distal portion of the ungual are preserved. The ungual is pointed, as in digit I. Digit III is represented only by an incomplete distal phalanx which is wider than the phalanges of digits I and II. The two distal phalanges are missing and only the distal part of the ungual is preserved. Digit IV lacks the proximal phalanx and ungual, and the distal non-ungual phalanx is poorly preserved. This phalanx is also wide, but shorter than the phalanx of digit III. The only remaining phalanx of digit V is poorly preserved, but is slender similar to the phalanges of digit II. The ungual of digit V is missing.

The phalanges of ZPAL MgD-II/3 are more slender than those of small adult *Protoceratops andrewsi* (e.g., NHMW 2015/0404/0001; Figs. 10B–10D). The unguals are pointed rather than rounded as reported by Brown & Schlaikjer (1940; Fig. 10E). Based on adult *Protoceratops andrewsi* specimens (e.g., small adults NHMW 2015/0404/0001, PIN 3143/7, large adults AMNH 6417, and 6467) it seems that this dinosaur had pointed, triangular manual unguals during a greater part of its life (Figs. 10C and 10D). The shape of the unguals and the presence of the lateral and medial neurovascular grooves placed laterally are also characters shared with *Auroraceratops* (Morschhauser, 2012). However, the unguals of *Protoceratops* differ from those of *Auroraceratops* in being flat rather than curved. Claw-like unguals occur in *Montanoceratops* (Chinnery & Weishampel, 1998) and *Leptoceratops* (AMNH 5205). They are more slender in *Yinlong* (Xu et al., 2006; Han et al., 2017) and *Psittacosaurus mongoliensis* (AMNH 6260). All non-ceratopsid members of Ceratopsia had pointed and narrow unguals of the manus (being only about as wide as the previous phalanx) during all their life (Fig. 10).

### Morphology of the pelvic girdle and hind limb

#### Ilium

**Figure 11:**
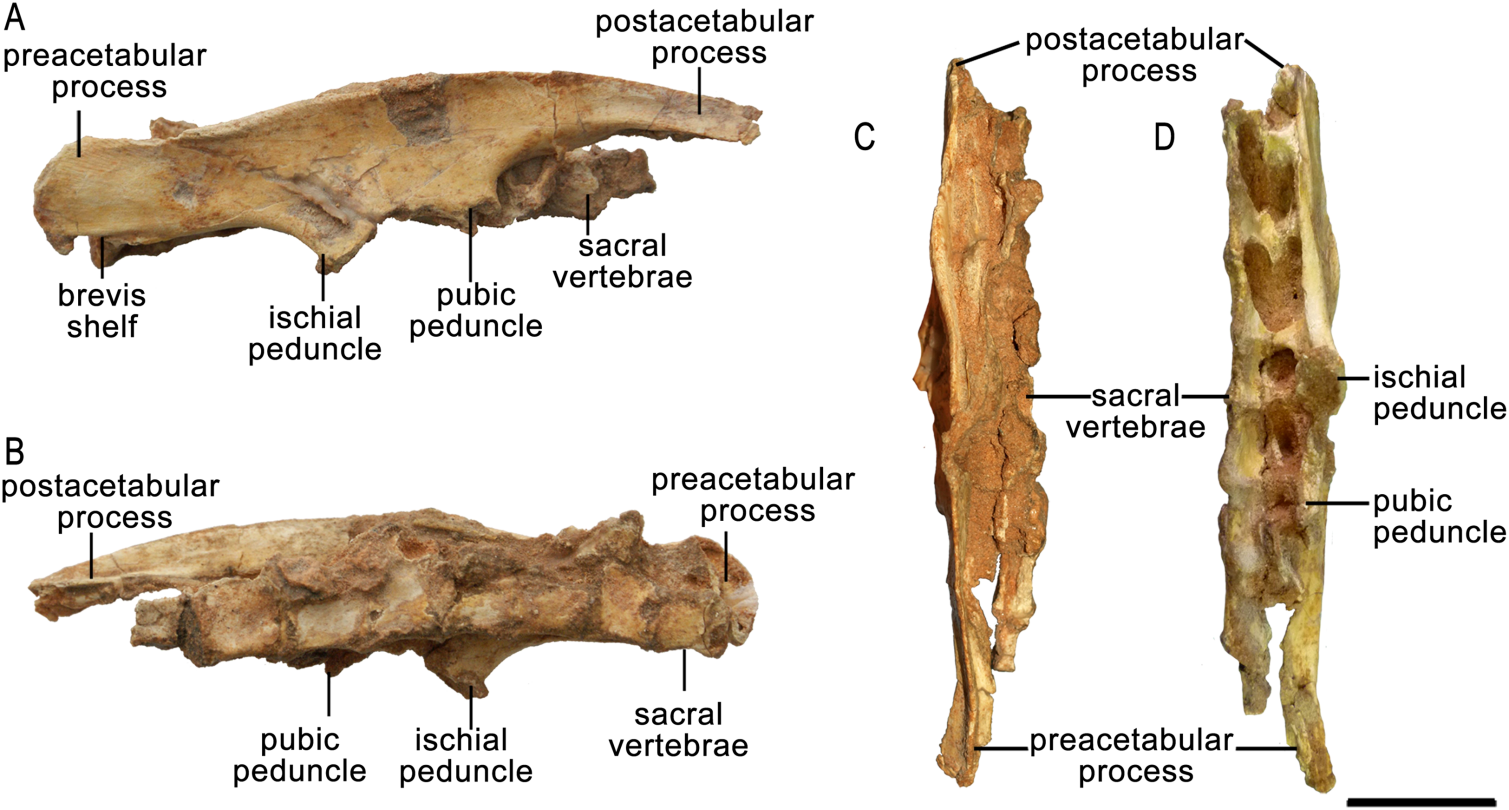
Right ilium of *Protoceratops andrewsi* ZPAL MgD-II/3 in (A) lateral, (B) medial, (C) dorsal, and (D) ventral views.

**Figure 12:**
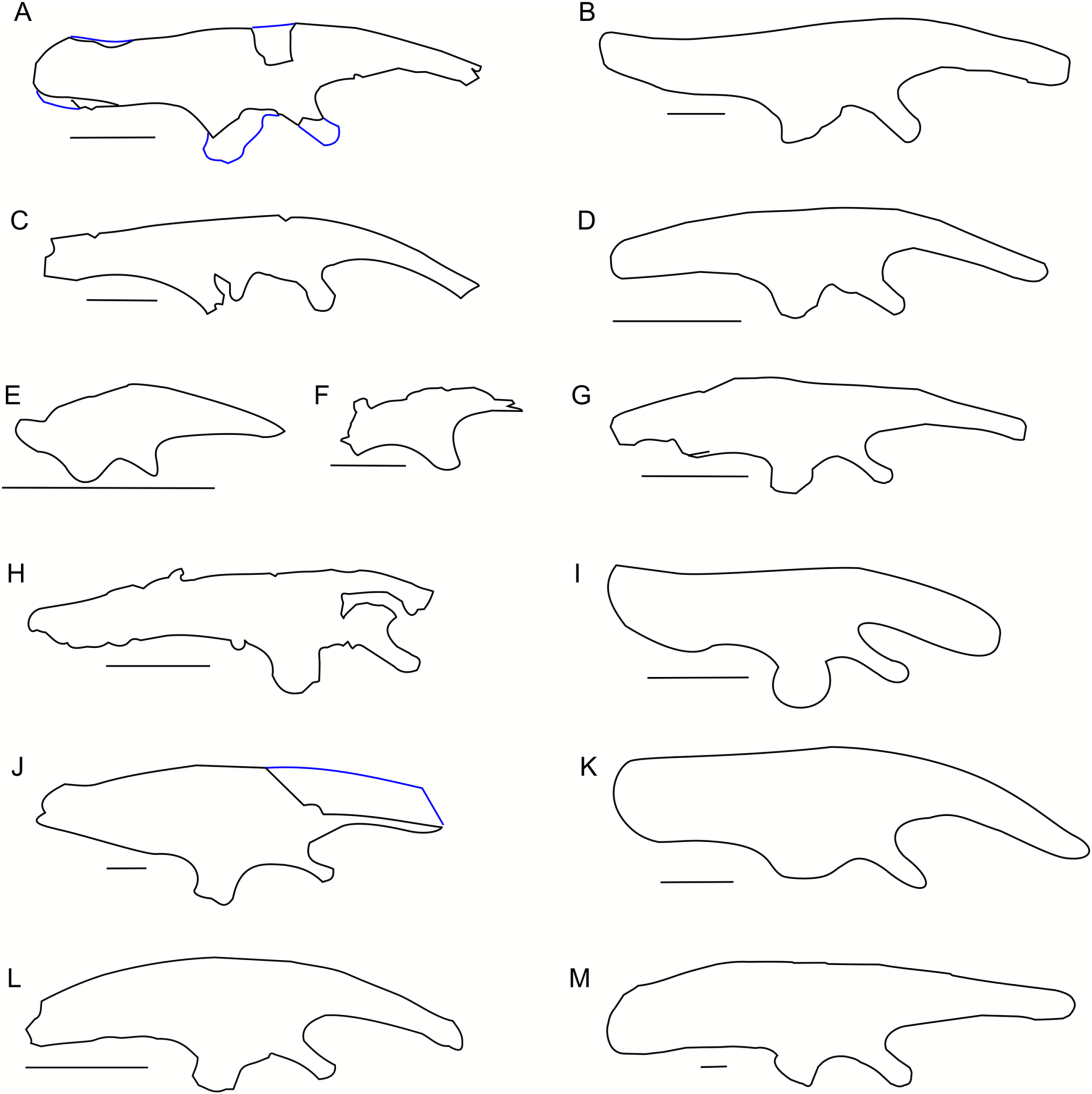
Ilium morphology. Right ilium outline in basal Ceratopsia. (A) subadult *Protoceratops andrewsi* ZPAL MgD-II/3; (B) adult *Protoceratops andrewsi* AMNH 6424; (C) *Yinlong downsi* IVPP V14530; (D) *Psittacosaurus mongoliensis* NHMW 1998z0064/0001; (E) *Breviceratops kolzowskii* ZPAL MgD-I/117; (F) *Bagaceratops rozhdestvenskyi* ZPAL MgD-I/155; (G) *Archaeoceratops oshimai* IVPP V 11115; (H) *Yamaceratops dorngobiensis* IGM 100/1315; (I) *Mosaiceratops azumai* ZMNH M8856; (J) *Ischioceratops zhuchengensis* ZCDM VOO16; (K) *Leptoceratops gracilis* NCM 8887; (L) *Auroraceratops rugosus* GSGM (07)9-49; (M) *Montanoceratops cerorhynchus* AMNH 5464. Missing elements are reconstructed with blue line. Scale bar: three cm. Outlined from: Xu et al. (2006) (C), Makovicky & Norell (2006) (H), He et al. (2015) (J), Sternberg (1951) (K), Morschhauser (2012) (L), based on You & Dodson (2003) (G), and Zheng, Jin & Xu (2015) (I).

Only the right ilium is preserved in ZPAL MgD-II/3, and it is fused to the sacrum (Fig. 11; Data S2). The overall shape of the ilium is characteristically ornithischian (He et al., 2015); the pre- and postacetabular portions are elongate, the preacetabular portion tapers cranially, and the dorsal margin of the ilium is dorsally convex (see small adults AMNH 6418, 6453, and 6470, large adults AMNH 6417 and 6467, and old individuals AMNH 6424 and 6466; Fig. 12B). In ZPAL MgD-II/3, the preacetabular part of the ilium is nearly the same length as the postacetabular portion. The preacetabular process curves ventrally toward its cranial end. The dorsal margin of the cranial portion of the ilium curves cranioventrally from the base of the preacetabular process, but the curvature is less pronounced than in adult *Protoceratops andrewsi* (see AMNH 6417, 6418, 6424, 6453, 6466, 6467, and 6470).

The lateral surface of the preacetabular process faces somewhat ventrolaterally. It bears a longitudinal concavity for the origin of the m. iliofemoralis or m. puboischiofemoralis internus (see Bates et al., 2012). The postacetabular process extends caudally and is dorsoventrally broader than the preacetabular portion of the ilium. The caudal end is convex, and its dorsal margin is inclined slightly caudodorsally. In adult *Protoceratops andrewsi* the distal end is narrower dorsoventrally and strongly pointed upward (e.g., AMNH 6417, 6418, 6424, 6453, 6466, 6467, and 6470). The brevis shelf of ventral part of the postacetabular process is pronounced in all studied *Protoceratops* individuals.

In dorsal view, the margin of the ilium is slightly S-shaped (Fig. 11C). The dorsal margin is elevated and slightly laterally everted in its central part to border the concavity for the origin of the main portion of the iliofemoralis, above the acetabulum. The acetabulum is not well preserved. The pubic peduncle is mediolaterally thickened and extends cranioventrally, but the distal end is missing. The ischial peduncle is transversely thickened and extends caudoventrally.

There are only slight differences in iliac morphology between subadult ZPAL MgD-II/3 and adult *Protoceratops andrewsi* specimens (e.g., AMNH 6417, 6418, 6424, 6453, 6466, 6467, and 6470; Figs. 12A and 12B). The ilia of *Yinlong* (Xu et al., 2006; Han et al., 2017; Fig. 12C) and *Yamaceratops* (Makovicky & Norell, 2006; Fig. 12H) are much shorter dorsoventrally, whereas the ilia of *Psittacosaurus mongoliensis* (NHMW 1998z0064/0001, AMNH 6534; Fig. 12D), *Auroraceratops* (Morschhauser, 2012; Fig. 12L), and *Archaeoceratops* (You, Tanoue & Dodson, 2010; Fig. 12G) are taller dorsoventrally than those of *Protoceratops*. In *Montanoceratops* (AMNH 5464; Fig. 12M) the dorsal margin of the ilium is straight. This is also true of *Ischioceratops*, in which the ilium is even taller dorsoventrally and has a dorsoventrally tall preacetabular process (He et al., 2015; Fig. 12J). In *Mosaiceratops* the postacetabular and preacetabular processes are both wide, and are pointed upward and downward, respectively (Zheng, Jin & Xu, 2015; Fig. 12I), whereas in *Leptoceratops* the preacetabular process is erect dorsally (Sternberg, 1951; Fig. 12K). The ilium of Leptoceratopsidae has a straight dorsal margin, and the postacetabular and preacetabular processes are wider dorsoventrally than seen in *Protoceratops andrewsi*. Also, the acetabulum is shallower, and the pubic peduncle more robust (Brown & Schlaikjer, 1942). In all non-ceratopsid Neoceratopsia the pubic peduncle is narrow and projects craniolaterally, whereas the ischial peduncle is wide transversely and craniocaudally (Fig. 12).

#### Ischium

**Figure 13:**
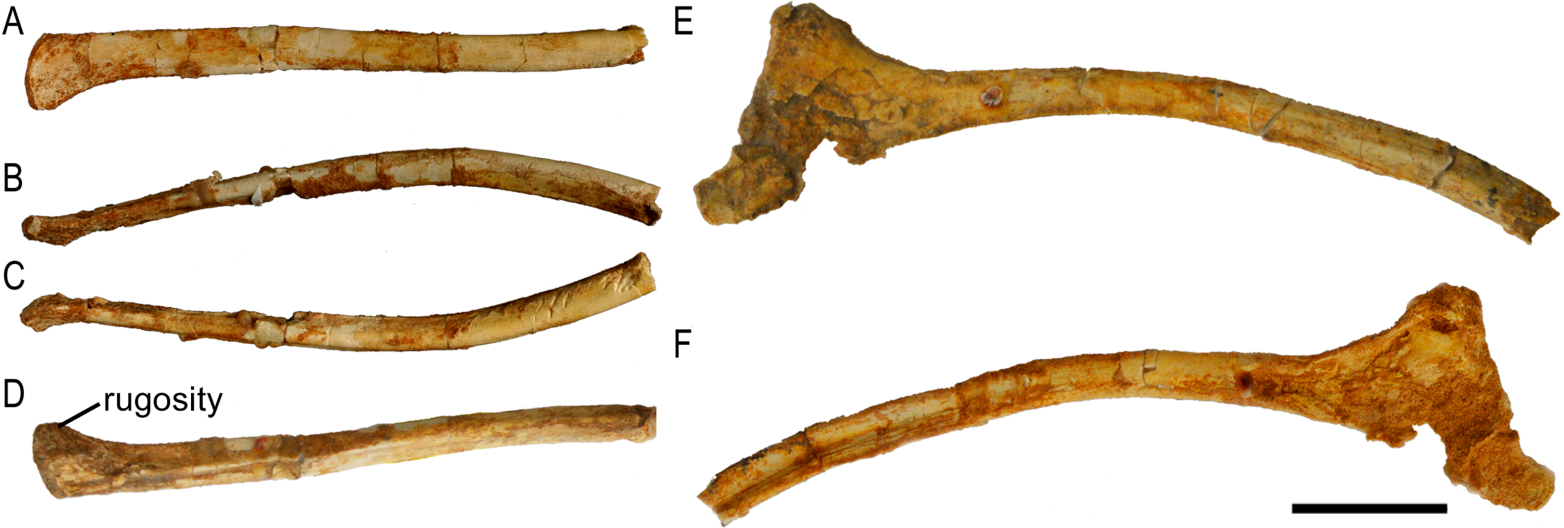
Ischia of *Protoceratops andrewsi* ZPAL MgD-II/3. A–D, Distal end of left ischium in (A) lateral, (B) dorsal, (C) ventral, and (D) medial views. E–F, Proximal part of right ischium in (E) lateral and (F) medial views. Scale bar: three cm.

The left ischium of ZPAL MgD-II/3 is more complete than the right one (Fig. 13), but its distal end is missing, and it lost its natural lateral curvature as a consequence of bilateral flattening and crushing. The proximal end with its medial surface damaged is subtriangular, and the cranial (i.e., acetabular) border shows a probably naturally formed concavity. The dorsal corner (the iliac peduncle) is broken. The right ischium, which lacks the proximal part, corresponds in both curvature and morphology to the shaft of the left ischium. It also retains its distal end (Fig. 13).

In lateral view the ischium is elongate and slender. Beyond the proximal end, the proximal part of the shaft is slightly deflected upward, whereas the middle part curves downward. As shown by the right ischium, the shaft is concave medially along its length and is rounded in cross-section. The distal end widens dorsoventrally and is bilaterally flattened, as well as curves medially to contact the distal end of its counterpart. A rugosity on its medial surface suggests a short symphysis. This part of the shaft bears a shallow groove bordered ventrally by a ridge extending along the medial surface of the ischium down to the distal end.

**Figure 14:**
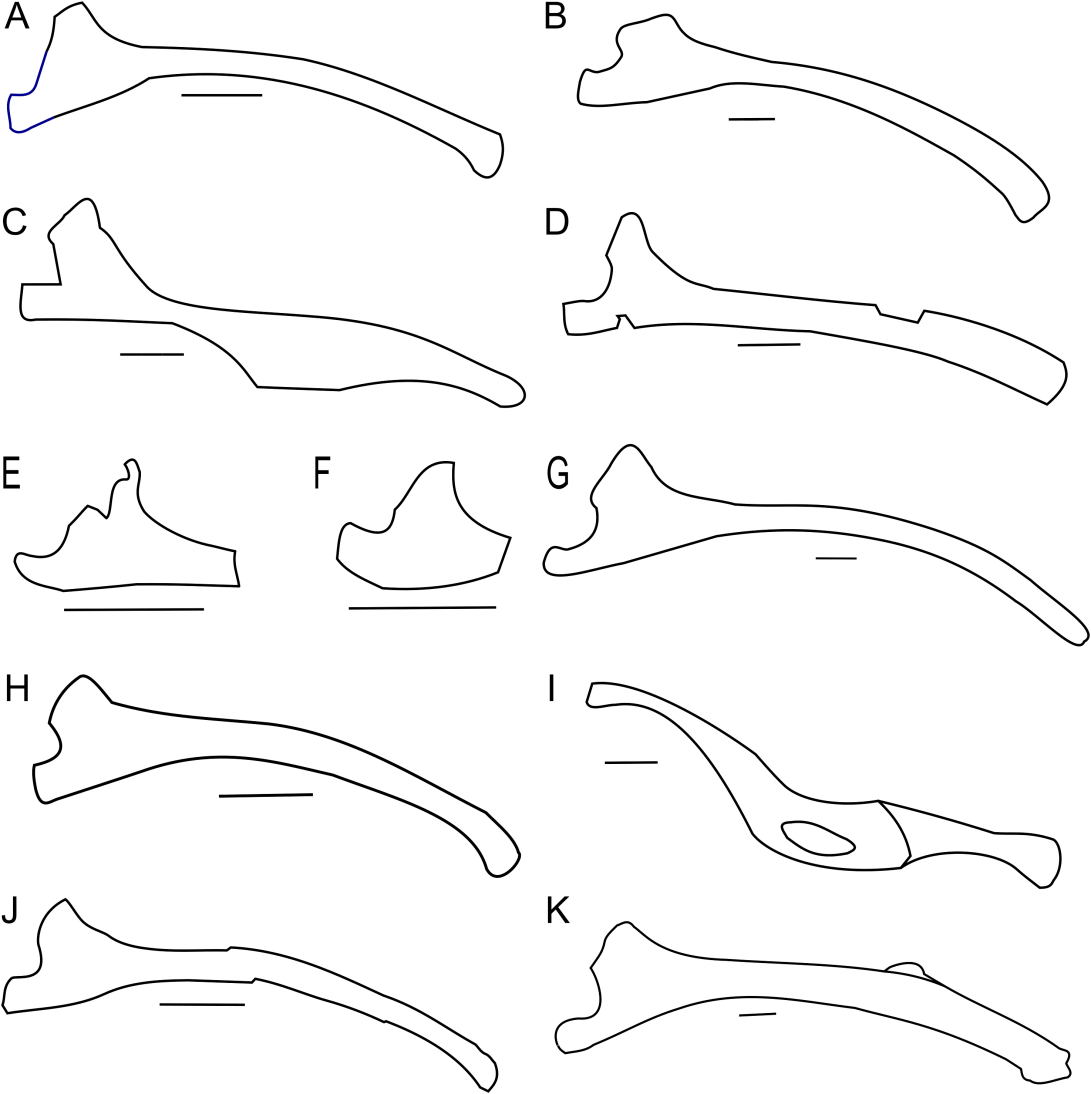
Ischium morphology. Right ischium outline in basal Ceratopsia. (A) Subadult *Protoceratops andrewsi* ZPAL MgD-II/3; (B) adult *P. andrewsi* AMNH 6417; (C) *Yinlong downsi* IVPP V14530; (D) *Psittacosaurus sibiricus* PM TGU 16/1-51; (E) *Bagaceratops rozhdestvenskyi* ZPAL MgD-I/142; (F) *Graciliceratops mongoliensis* ZPAL MgD-I/156; (G) *Leptoceratops gracilis* CMN 8887; (H) *Mosaiceratops azumai* ZMNH M8856; (I) *Ischioceratops zhuchengensis* ZCDM VOO16; (J) *Auroraceratops rugosus* GSGM (09)06; (K) *Montanoceratops cerorhynchus* AMNH 5464. Missing elements are reconstructed with blue line. Scale bar: three cm. Modified from: Han et al. (2017) (C), and outlined from: Averianov et al. (2006) (D), Sternberg (1951) (G), Zheng, Jin & Xu (2015) (H), He et al. (2015) (I), and Morschhauser (2012) (J).

The general morphology of the ischia of ZPAL MgD-II/3 is the same as in adult *Protoceratops andrewsi* specimens (e.g., AMNH 6417 and 6424; Fig. 14B) and is characterized by a shaft that is slender, long and rounded in cross-section, with an expanded distal end (Figs. 14A and 14B). The ischial curvature of *Protoceratops andrewsi* is about 10° (Adams, 1987). Although it is impossible to measure the curvature of ZPAL MgD-II/3, the morphology of the right ischium conforms to that of other *Protoceratops* ischia (e.g., large adult AMNH 6417 and old AMNH 6424). In *Psittacosaurus* the shaft of the ischium is straighter and more robust, and is dorsoventrally compressed and only slightly expanded at the end (Averianov et al., 2006) or not expanded at all (Russell & Zhao, 1996). The symphysis is less well known in *Psittacosaurus*; *Psittacosaurus mongoliensis* has a short symphysis (Sereno, 1987), but in *Psittacosaurus ordosensis* (Russell & Zhao, 1996) and *Psittacosaurus sibiricus* (Averianov et al., 2006) it is absent. The ischial shaft of Leptoceratopsidae is laterally compressed and robust (*Montanoceratops* (AMNH 5464; Fig. 14K), and *Leptoceratops* (Sternberg, 1951; Ostrom, 1978; Fig. 14G). In non-leptoceratopsid neoceratopsians such as *Mosaiceratops* (Zheng, Jin & Xu, 2015; Fig. 14H), *Auroraceratops* (Morschhauser, 2012; Fig. 14J), and *Koreaceratops* (Lee, Ryan & Kobayashi, 2011) the ischial shaft is oval in cross-section and more slender, as in ZPAL MgD-II/3. The slight lateral curvature of the ischium of ZPAL MgD-II/3 is similar to the condition found in *Mosaiceratops* (Zheng, Jin & Xu, 2015), differing from the straight ischial shaft of *Archaeoceratops* (You & Dodson, 2003) and *Koreaceratops* (Lee, Ryan & Kobayashi, 2011). On the other hand, in *Leptoceratops* (Sternberg, 1951; Ostrom, 1978) and *Montanoceratops* (AMNH 5464; Brown & Schlaikjer, 1942) the ischial shaft is more curved than in ZPAL MgD-II/3.

The ischial shaft of *Ischioceratops* is curved ventrally (not dorsally) and flattened laterally, bearing a fenestra in the middle (He et al., 2015). The ischium has an expanded distal end with a rugose surface in *Mosaiceratops* (Zheng, Jin & Xu, 2015), *Auroraceratops* (Morschhauser, 2012), *Ischioceratops* (He et al., 2015), and *Koreaceratops* (Lee, Ryan & Kobayashi, 2011). On the other hand, *Leptoceratops* (Sternberg, 1951; Ostrom, 1978) and *Montanoceratops* (AMNH 5464) lack any expansion of the distal end of the ischium (Fig. 14).

#### Femur

**Figure 15:**
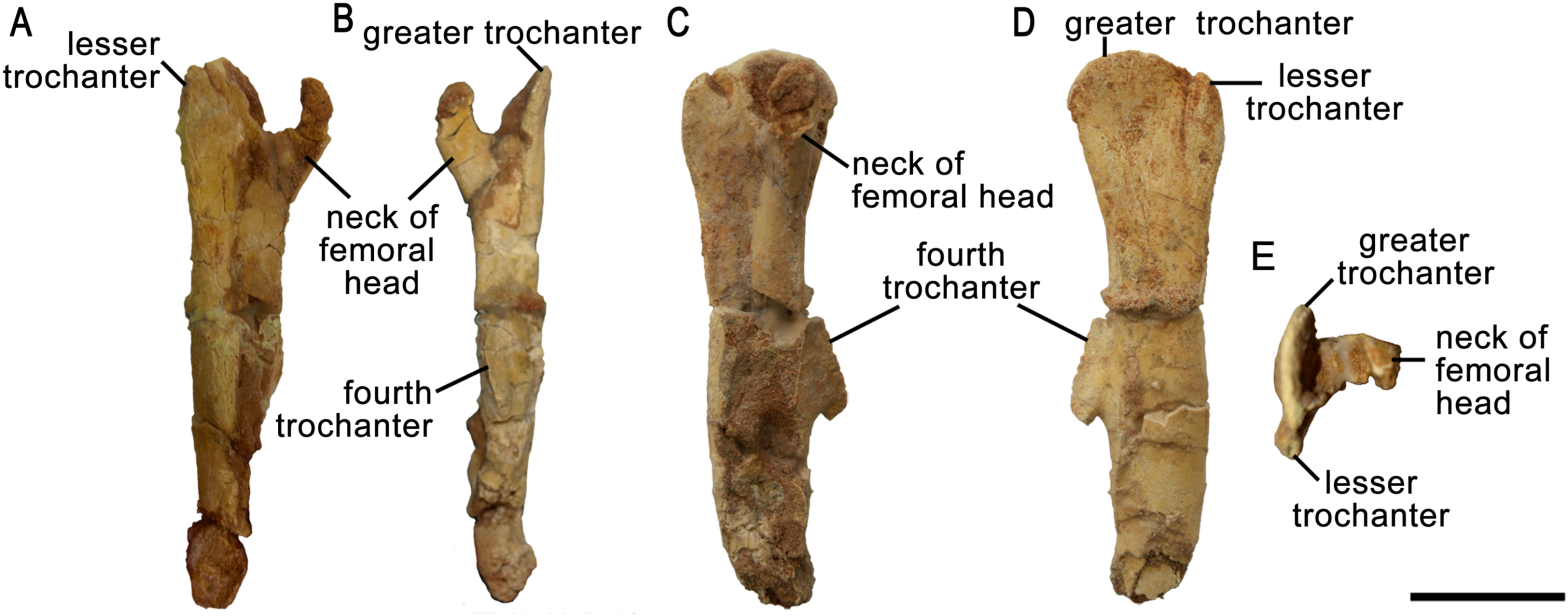
Right femur of *Protoceratops andrewsi* ZPAL MgD-II/3 in (A) cranial, (B) caudal, (C) medial, (D) lateral, and (E) dorsal views.

**Figure 16:**
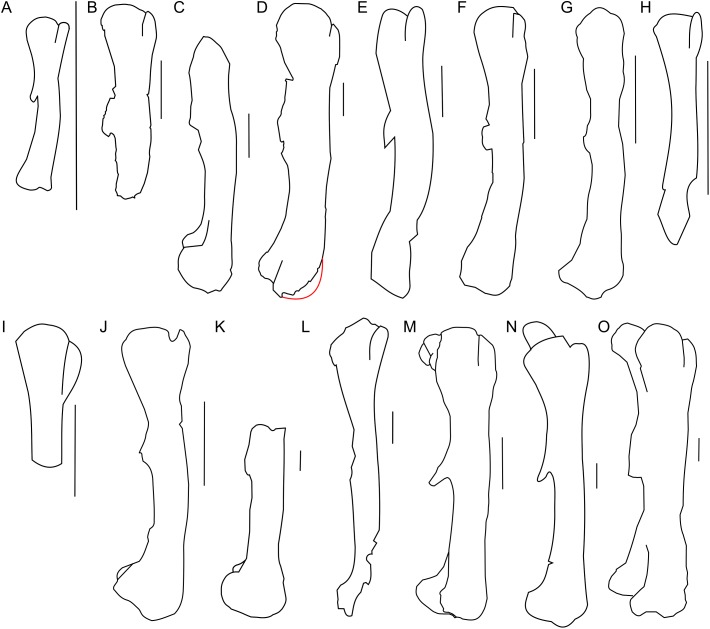
Femur morphology. Right femur outline in basal Ceratopsia, lateral view. (A) Hatchling of *Protoceratops andrewsi* MPCD-100/530; (B) subadult *P. andrewsi* ZPAL MgD-II/3; (C) subadult *P. andrewsi* AMNH 6418; (D) adult *P. andrewsi* AMNH 6424; (E) *Psittacosaurus mongoliensis* NHMW 1998z0064/0001; (F) *Yinlong downsi* IVPP V14530; (G) *Graciliceratops mongoliensis* ZPAL MgD-I/156; (H) *Breviceratops kozlowskii* ZPAL MgD-I/117; (I) *Bagaceratops rozhdestvenskyi* ZPAL MgD-I/142; (J) *Archaeoceratops yujingziensis* CASG-IG-VD-0003; (K) *Leptoceratops gracilis* AMNH 5205; (L) *Ischioceratops zhuchengensis* ZCDM VOO16; (M) *Auroraceratops rugosus*, GSGM (07)9-60; (N) *Cerasinops hodgskissi* MOR 300; (O) *Montanoceratops cerorhynchus* AMNH 5464. Missing elements are reconstructed with red line. Scale bar: one cm for (A) and three cm for (B–J). Outlined from: Fastovsky et al. (2011) (A), Han et al. (2017) (F), You, Tanoue & Dodson (2010) (J), He et al. (2015) (L), Morschhauser (2012) (M), and Chinnery & Horner (2007) (N).

Only a right incomplete femur of ZPAL MgD-II/3 is preserved. About one-fourth of the distal part is missing, as well as the most proximal portion (Fig. 15). In cranial view the femur is straight and consists of an elongate and slender shaft. During ontogeny the femur in general becomes more robust (Figs. 16A–16D).

The proximal end is expanded both transversely and craniocaudally. In cranial view the femoral head is separated from the greater trochanter by a notch. Because of the poor state of preservation, the exact depth of the notch is unknown, but the notch in subadults (e.g., ZPAL MgD-II/35) is generally deeper than in adult *Protoceratops andrewsi* (see large adults: AMNH 6417 and senile individuals AMNH 6424; Figs. 16C and 16D). In lateral view the lesser trochanter is separated from the greater trochanter only by a shallow cleft. The greater trochanter is expanded craniocaudally, and the lesser trochanter is reduced in size.

The fourth trochanter projects from the caudal surface at the level of the mid-length of the femoral shaft. It forms a transversely thin, pendent crest with a proximodistally wide base. The distal part of the fourth trochanter does not adhere to the shaft and is directed caudomedially. The space between a more projected dorsally greater trochanter and the femoral head gets shallower, the femur becomes straighter, and the fourth trochanter stays pendent in adult *Protoceratops andrewsi* (large adults: AMNH 6417, 6467, and old individual, AMNH 6424). The femur of the non-ceratopsid neoceratopsians, including *Yinlong* (Xu et al., 2006; Fig. 16F) and *Psittacosaurus mongoliensis* (AMNH 6534, 6541, and NHMW 1998z0064/0001; Fig. 16E) bears a pendent fourth trochanter and is caudally concave (Fig. 16). Because the distal end of the femur is missing in ZPAL MgD-II/3, there is no information about the femoral curvature. However, juvenile *Protoceratops* (Fastovsky et al., 2011) have an arched femur in lateral view, whereas adults have straight femora (small adult AMNH 6418, large adults: AMNH 6417, 6424, and 6467); in all of these specimens, regardless of ontogenetic age, the fourth trochanter stays thin and pendent. Among non-ceratopsid ceratopsians, only *Mosaiceratops* (Zheng, Jin & Xu, 2015), *Breviceratops* (ZPAL MgD-I/117; Fig. 16H), and *Archaeoceratops* (You, Tanoue & Dodson, 2010; Fig. 16J) have the femur concave caudally. The fourth trochanter is only slightly pendent in *Mosaiceratops*, and seems strongly pendent in *Archaeoceratops* (You, Tanoue & Dodson, 2010; Zheng, Jin & Xu, 2015). The fourth trochanter is poorly preserved in *Breviceratops* (ZPAL MgD-I/117) and also in *Graciliceratops* (ZPAL MgD-I/156). A strongly pendent fourth trochanter occurs in *Auroraceratops* and *Cerasinops*, but in the latter it is directed more ventrally (Chinnery & Horner, 2007; Morschhauser, 2012; Figs. 16M and 16N). The femoral shaft in both taxa is straight, and the femoral head is directed slightly caudally. These features are also seen in *Montanoceratops*, but in this taxon the fourth trochanter is more robust (as noticed also by Makovicky, 2010) and only slightly pendent (AMNH 5464; Fig. 16O). A similar condition of the fourth trochanter can be seen in *Ischioceratops*, but the femoral shaft is slightly arched in lateral view (He et al., 2015; Fig. 16L). The lesser trochanter of *Yinlong* is only slightly smaller than the greater trochanter (Xu et al., 2006; Fig. 16F), whereas in other non-ceratopsid ceratopsians it becomes much wider craniocaudally than the lesser trochanter. In *Bagaceratops* (ZPAL MgD-I/142; Fig. 16I), however, the lesser trochanter is wide craniocaudally in comparison to the condition in other non-ceratopsid ceratopsians. In *Breviceratops* (ZPAL MgD-I/117), the lesser trochanter is higher dorsally than the greater trochanter, as in *Yinlong* (Xu et al., 2006). In all non-ceratopsid ceratopsians (with the exception of *Archaeoceratops*) the lesser trochanter is closely appressed to the greater trochanter. The lesser trochanter of *Protoceratops andrewsi* is less expanded craniocaudally than in other non-ceratopsid ceratopsians, with the possible exception of *Graciliceratops*, in which the lesser trochanter is poorly preserved (ZPAL MgD-I/156; Fig. 16G). In younger *Protoceratops andrewsi* (MPC-D 100/530, ZPAL MgD-II/3), the difference between the greater and lesser trochanter is smaller than in adults (e.g., old AMNH 6424). The distal condyles are bulbous in all non-ceratopsid neoceratopsians.

#### Tibia

**Figure 17:**
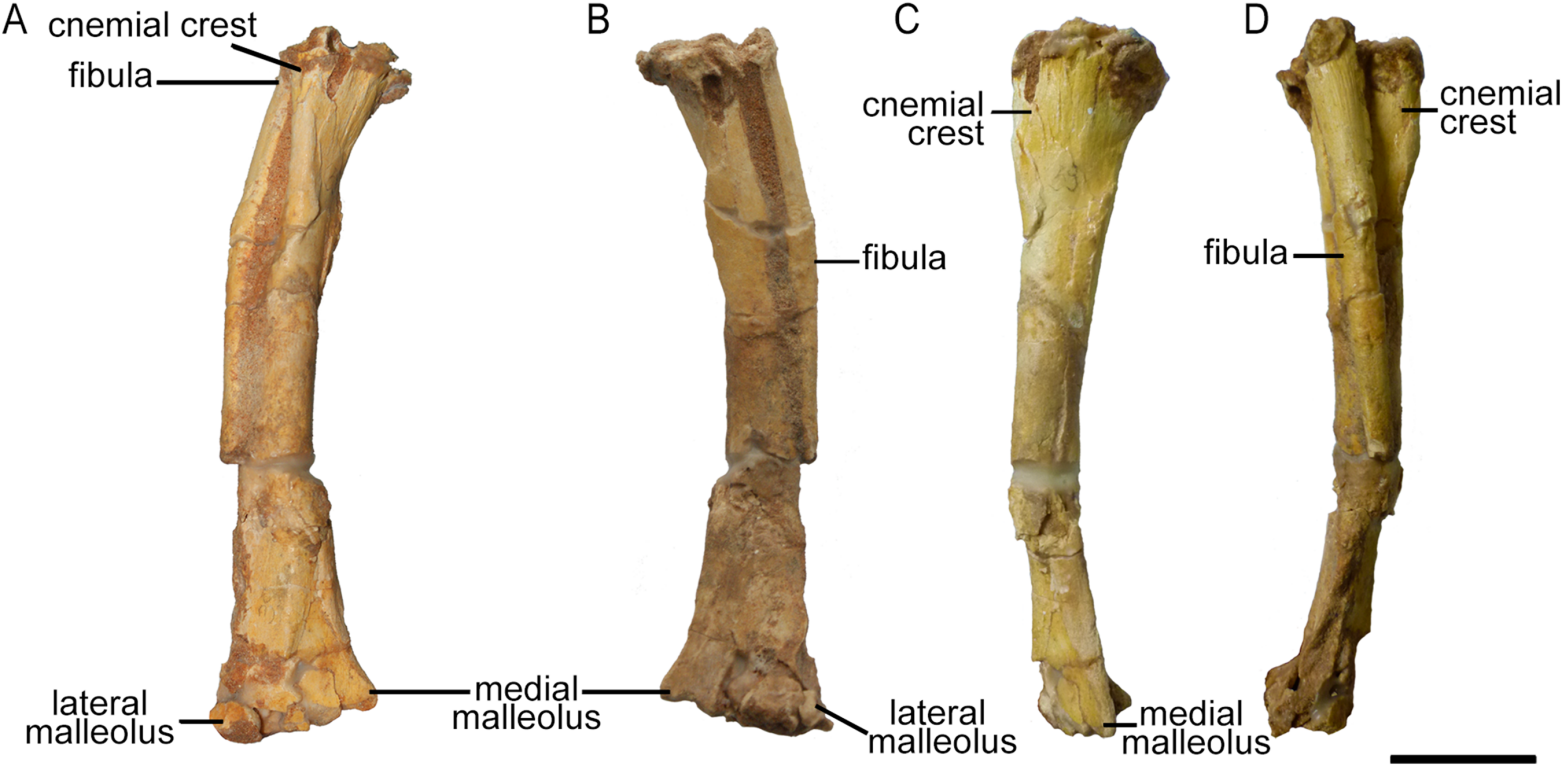
Right tibia and fibula of *Protoceratops andrewsi* ZPAL MgD-II/3 in (A) cranial, (B) caudal, (C) medial, and (D) lateral view.

Only the right tibia is preserved in ZPAL MgD-II/3, and this bone is almost complete and preserved in articulation with the fibula. However, the proximal parts of both bones have been bent medially as a result of crushing, affecting about one-fourth of their lengths (Fig. 17). On the proximal tibia, evidence of larval foraging (i.e., characteristic rounded hollows), similar to that described by Kirkland & Bader (2010) can be seen.

**Figure 18:**
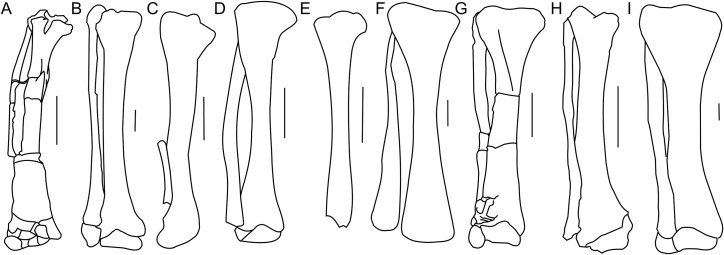
Tibia and fibula morphology. Right tibia and fibula outlines in basal Ceratopsia, cranial view. (A) Subadult *Protoceratops andrewsi* ZPAL MgD-II/3; (B) adult *Protoceratops andrewsi* AMNH 6424; (C) *Yinlong downsi* IVPP V14530; (D) *Psittacosaurus mongoliensis* NHMW 1998z0064/0001; (E) *Graciliceratops mongoliensis* ZPAL MgD-I/156; (F) *Leptoceratops gracilis* CMN 8887; (G) *Auroraceratops rugosus* GSGM (07)22; (H) *Cerasinops hodgskissi* MOR 300; (I) *Montanoceratops cerorhynchus* AMNH 5464. Scale bar: three cm. Outlined from: Han et al. (2017) (C), Sternberg (1951) (F), Morschhauser (2012) (G), and Chinnery & Horner (2007) (H).

The tibia is expanded craniocaudally at its proximal end, but the shaft then narrows to an oval cross-section before expanding transversely at the distal end as in all dinosaurs (Weishampel, Dodson & Osmólska, 2004). The shaft of the tibia most probably was straight, as in all *Protoceratops andrewsi* specimens; the current curved state of the shaft of ZPAL MgD-II/3 (Fig. 17) is an artifact caused by damage (see Fig. S6). The tibia of ZPAL MgD-II/3 is more slender than in mature *Protoceratops andrewsi* (Fig. 18B).

The cnemial crest is a ridge that extends along the cranial face of the bone from its proximal end down to approximately one-fourth of shaft length. The ridge curves slightly laterally. Under the cnemial crest, at the medial surface of the shaft a convex structure is located. The fibular crest is not visible. At the distal end of the tibia, the medial malleolus is flattened craniocaudally but prominent medially. The lateral malleolus is bigger than the medial malleolus and is expanded craniocaudally, as in all ceratopsians. The lateral surface of the lateral malleolus bears an articulation surface for the fibula. The proximal margin of the distal malleoli is sinusoidal in cranial view.

The tibia of adult *Protoceratops andrewsi* is similar to ZPAL MgD-II/3 in general shape, but the cnemial crest seems to be more curved laterally. The proximal condyles and distal malleoli are poorly preserved in ZPAL MgD-II/3 so it is difficult to compare them to those of adult *Protoceratops andrewsi* (e.g., adult AMNH 6424; Fig. 18B). The tibia is more slender in *Psittacosaurus mongoliensis* (AMNH 6254, NHMW 1998z0064/0001; Fig. 18D) and *Graciliceratops* (ZPAL MgD-I/156; Fig. 18E), in both of which it is also wider proximally than distally, because the cnemial crest and the medial surface of the proximal part of the tibial shaft are expanded in the lateral and medial directions, respectively. Similar expansion of the proximal part of the tibia can also be seen in *Auroraceratops* (Morschhauser, 2012; Fig. 18G), *Leptoceratops* (Sternberg, 1951; Fig. 18F), and *Montanoceratops* (AMNH 5464; Fig. 18I), in which the tibia is more robust. In *Cerasinops* (Gilmore, 1939; Chinnery & Horner, 2007; Fig. 18H) the distal end is more expanded transversely than the proximal one, and the cnemial crest is also less wide. The proximal and distal ends of the tibia are similar in transverse width in *Protoceratops* (e.g., ‘old’ AMNH 6424) and *Ischioceratops* (He et al., 2015). The distal end of the tibia angles medially in all described non-ceratopsid ceratopsians, with the exception of *Auroraceratops* (Morschhauser, 2012) and *Ischioceratops* (He et al., 2015). The medial inclination is slight in *Psittacosaurus mongoliensis* (AMNH 6254, and NHMW 1998z0064/0001) and *Montanoceratops* (AMNH 5464), but stronger in *Koreaceratops* (Lee, Ryan & Kobayashi, 2011), *Leptoceratops* (AMNH 5205), *Protoceratops* (AMNH 6424), and *Cerasinops* (Gilmore, 1939; Chinnery & Horner, 2007; Fig. 18).

#### Fibula

The right fibula of ZPAL MgD-II/3 is preserved in articulation with the tibia. The proximal end of the bone is damaged, and approximately the distal one-third of the bone is missing (Fig. 17). The fibula of ZPAL MgD-II/3 is similar in shape to that of adult *Protoceratops andrewsi* (e.g., AMNH 6424; Fig. 18B).

In general the fibula of *Protoceratops* is straight and slender, similar to the condition of *Ischioceratops* (He et al., 2015), *Cerasinops* (Gilmore, 1939; Chinnery & Horner, 2007; Fig. 18H), and *Auroraceratops* (Morschhauser, 2012; Fig. 18G). In lateral view the proximal part of the fibula is expanded craniocaudally, but more distally the fibula becomes constricted. On the other hand, the fibula of *Montanoceratops* (AMNH 5464; Fig. 18I) is more robust, but also straight, and more gracile than the fibulae in all ceratopsids. In non-ceratopsid ceratopsians the proximal end of the curved fibula contacts the caudolateral part of the tibia whereas the distal part contacts the craniolateral surface of the distal end of the tibia above the calcaneus (You & Dodson, 2004).

#### Pes

**Figure 19:**
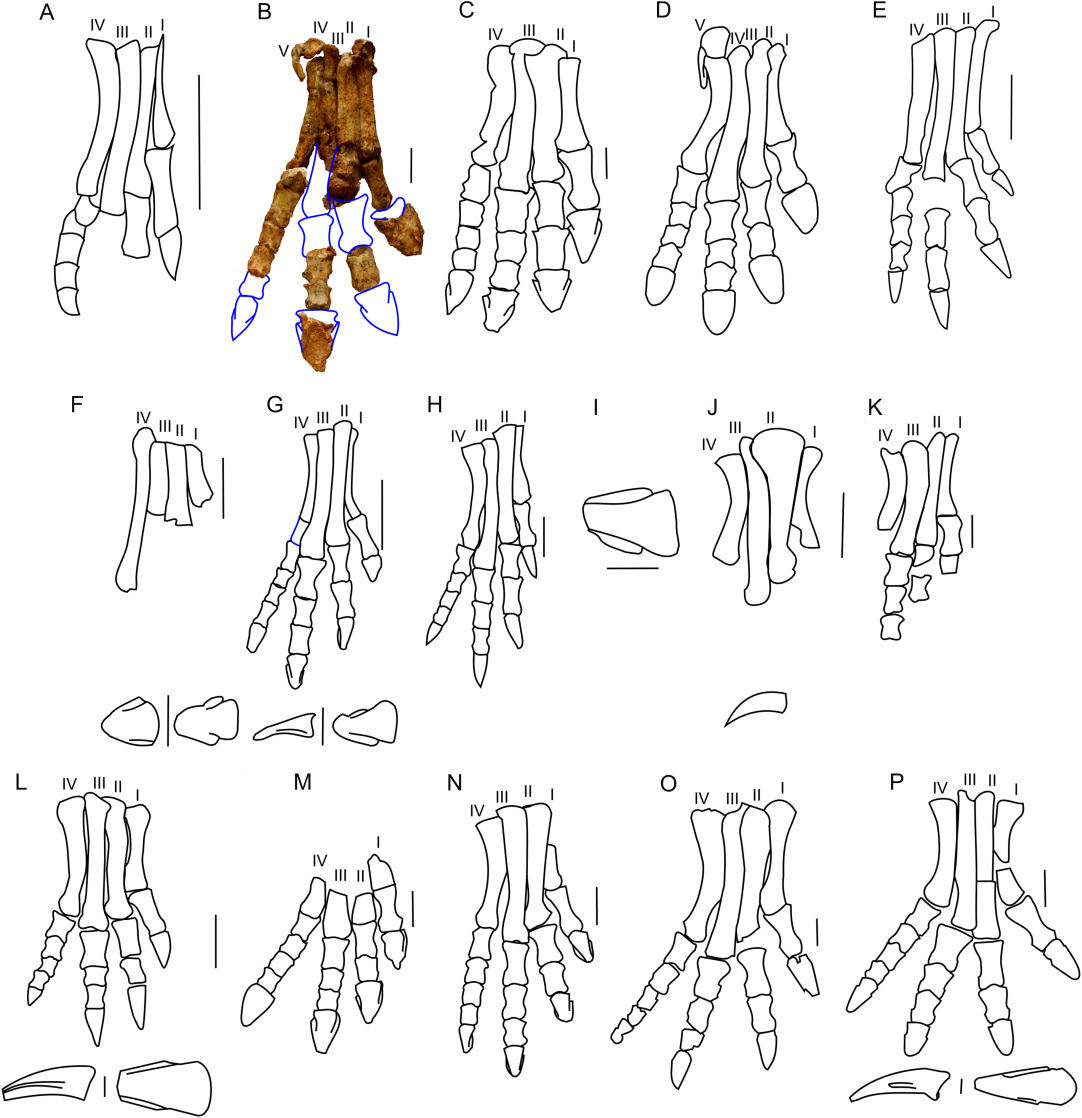
Pes morphology. Right pes outline in basal Ceratopsia. (A) Hatchling of *Protoceratops andrewsi* MPCD-100/530; (B) subadult *P. andrewsi* ZPAL MgD-II/3; (C) adult *P. andrewsi* AMNH 6418; (D) adult *P. andrewsi* AMNH 5351; (E) pedal unguals of *Yinlong downsi* IVPP V14530; (F) *Bagaceratops rozhdestvenskyi*, pes (ZPAL MgD-I/320) and pedal unguals (PIN 614/53); (G) *Graciliceratops mongoliensis* ZPAL MgD-I/156; (H) *Archaeoceratops oshimai* IVPP V11115; (I) *Yamaceratops dorngobiensis* IGM 100/1303; (J) metatarsals and pedal claw of *Mosaiceratops azumai* ZMNH M8856; (K) metatarsals, some phalanges and fragmentary ungual of digit I of *Koreaceratops hwaseongensis* KIGAM VP 200801; (L) pes of *Leptoceratops gracilis* CMN 8887 and pedal claw AMNH 5205 in lateral (left) and dorsal (right) view; (M) distal part of metatarsals and pedal phalanges of *Udanoceratops tschizhovi* PIN 4046/11; (N) *Auroraceratops rugosus* GSGM (07)9-39; (O) *Cerasinops hodgskissi* MOR 300; (P) pes of *Montanoceratops cerorhynchus* MOR 542 and pedal claws of AMNH 5464 in lateral (above) and dorsal (below) views. Scale bar: three cm for pes (A–D, F, H–O) and one cm for pedal claws in (E, F, J, L and P). Outlined from: Fastovsky et al. (2011) (A), Brown & Schlaikjer (1940) (D), Xu et al. (2006) (E), Tereshchenko (2008) (F and M), Makovicky & Norell (2006) (I), Zheng, Jin & Xu (2015) (J), Lee, Ryan & Kobayashi (2011) (K), Sternberg (1951) (L), Morschhauser (2012) (N), Chinnery & Horner (2007) (O), and Chinnery & Weishampel (1998) (P) and based on You & Dodson (2003) (H).

The astragalus and calcaneus are both missing. All metatarsals (I–V) of the right foot of ZPAL MgD-II/3 are preserved, and metatarsals I–IV are articulated (Fig. 19). Metatarsal I, which is the best preserved of the metatarsals and remains in articulation with the first phalanx, seems to be the shortest metatarsal other than the disarticulated metatarsal V. The relative lengths of the metatarsals may only be estimated with reference to the adult specimen of *Protoceratops andrewsi* (AMNH 6424) described and figured by Brown & Schlaikjer (1940), in which metatarsal III is the longest of all and metatarsal V is the shortest. The proximal parts of the metatarsals are closely pressed against each other. As a whole, the metatarsus is arched, producing a concave plantar surface that probably represents a natural life position. The proximal end of metatarsal II is transversely expanded. The distal ends of metatarsals I and II are rather flattened in the craniocaudal direction, and are transversely expanded. The pedal phalanges are disarticulated, and only some of them have been preserved.

The pes of ZPAL MgD-II/3 is very slender, and the metatarsals are closely pressed against each other (Fig. 19B). During the ontogeny of *Protoceratops andrewsi* the pes becomes more robust and wider transversely. In senile *Protoceratops andrewsi* (e.g., ZPAL MgD-II/11) the metatarsals are more flattened craniocaudally than in younger individuals. All non-ceratopsid ceratopsians have a slender pes and compacted metatarsals (You & Dodson, 2004). This is contrary to the evolutionary trend observed in Ceratopsidae, in which the metatarsal segment is less compacted, and the phalanges and metatarsals are flattened and shortened (Dodson, Forster & Sampson, 2004).

The proximal end of metatarsal I is crushed and bears a dorsal prominence that probably represents a tubercle for the insertion of the extensor muscle. The planto-medial surface is strongly concave. In plantar view, the second metatarsal overlaps the first one obliquely (the line of contact trending from mediodorsal to lateroplantar), which seems to be a natural contact between the metatarsals. The distal end is missing, and the distal portion of the bone is craniocaudally expanded.

Metatarsal II is the most massive of all the metatarsals. Its proximal end is missing, but a small dorsal protrusion is a plausible remnant of an extensor tubercle. The distal end of the bone is crushed. Metatarsal II is clearly longer than metatarsal I. A crest extending along the lateral side of the dorsal surface of metatarsal II borders a surface for metatarsal III. Distally metatarsal II is in articulation with the remnants of the first phalanx.

Only the shaft (which is generally straight) of metatarsal III is preserved. The proximal end of the bone is sub-circular in cross-section. The articular facet for the tarsus descends obliquely to the plantar side, where it turns into a distinct plantar crest.

Metatarsal IV is the most complete of all the metatarsals. The shaft is laterally concave. The distal part of metatarsal IV is laterally bent. The distal end is expanded mediolaterally and bears a deep midline groove, which crosses the distal surface from the cranial to the plantar side. Metatarsal IV remains in articulation with the first phalanx, which is preserved.

Metatarsal V is reduced as in all non-ceratopsid neoceratopsians and adheres to the proximal part of metatarsal IV. Metatarsal V is short and paddle-shaped, and its proximal end is craniocaudally flattened. The shaft is expanded craniocaudally and compressed transversely.

*Protoceratops andrewsi* has the phalangeal formula of 2-3-4-5-0, which is typical of Ceratopsia (Dodson, Forster & Sampson, 2004; You & Dodson, 2004). However, the pes of ZPAL MgD-II/3 is incomplete (Fig. 19B). The phalanges of digits I, IV, and partly of digit II, are articulated (Fig. 19B). Most of the phalanges are longer than wide, the condition characteristic of non-ceratopsid Neoceratopsia (Morschhauser, 2012). The proximal phalanges do not bear dorsal processes proximally. However, the subsequent phalanges have pronounced dorsal processes that are continuous with the midline ridges, dividing the proximal articular surfaces of the phalanges into two concave areas for the reception of the condyle of the previous phalanx.

Digit I is represented by a single proximal phalanx in addition to the ungual phalanx. The proximal phalanx is the longest phalanx of the foot, and has a flat dorsal surface and concave plantar surface. Also, it is more concave at the medial than at the lateral margin. The distal and proximal parts are transversely expanded. The ungual of digit I is wide mediolaterally and flattened transversely. Its proximal margin is concave, and the lateral and medial margins are slightly convex; the ungual was probably slightly pointed, as in senile *Protoceratops andrewsi* (ZPAL MgD-II/11).

Digit II is represented only by a distal non-ungual phalanx. The proximal phalanx and the ungual are missing. The proximal and distal ends of the proximal phalanx are poorly preserved, and the phalanx has flattened dorsal and plantar faces.

Digit III retains an ungual and two preserved phalanges, most probably the second and third ones. The third phalanx is shorter and less concave laterally and medially, than the second. The ungual of digit III is longer and wider transversely than the phalanx. The plantar and dorsal surfaces are flattened, and the distal, medial, and lateral margins are damaged.

Only three articulated phalanges remain intact in digit IV. The distalmost non-ungual phalanx and the ungual are not preserved. The second and third phalanges are the shortest phalanges in the foot, whereas the first phalanx is almost twice as long as either of the distal phalanges.

The shape of the unguals varies among non-ceratopsid neoceratopsians. *Bagaceratops* and *Udanoceratops* have unguals similar to those of *Protoceratops* (ZPAL MgD-II/11) (Tereshchenko, 2008; Figs. 19A–19C, 19F and 19M). *Auroraceratops* has narrow, pointed, and short unguals, similar in length to the distalmost phalanges of the second and third digits (Morschhauser, 2012; Fig. 19N). However, non-ceratopsid Ceratopsia (*Yinlong* (Xu et al., 2006; Fig. 19E), *Hulianceratops* (Han et al., 2015), and *Psittacosaurus* (Sereno, 1987), and most non-ceratopsid Neoceratopsia (e.g., *Graciliceratops* ZPAL MgD-I/156; Fig. 19G), *Leptoceratops* (AMNH 5205; Fig. 19L), *Koreaceratops* (Lee, Ryan & Kobayashi, 2011; Fig. 19K), *Mosaiceratops* (Zheng, Jin & Xu, 2015; Fig. 19J), *Cerasinops* (Chinnery & Horner, 2007; Fig. 19O), *Montanoceratops* (AMNH 5464; Fig. 19P), *Prenoceratops* (Chinnery, 2004b), and *Archaeoceratops* (You & Dodson, 2003; Fig. 19H) have narrow, long, and pointed unguals.

### Hind limb bones ratio

**Figure 20:**
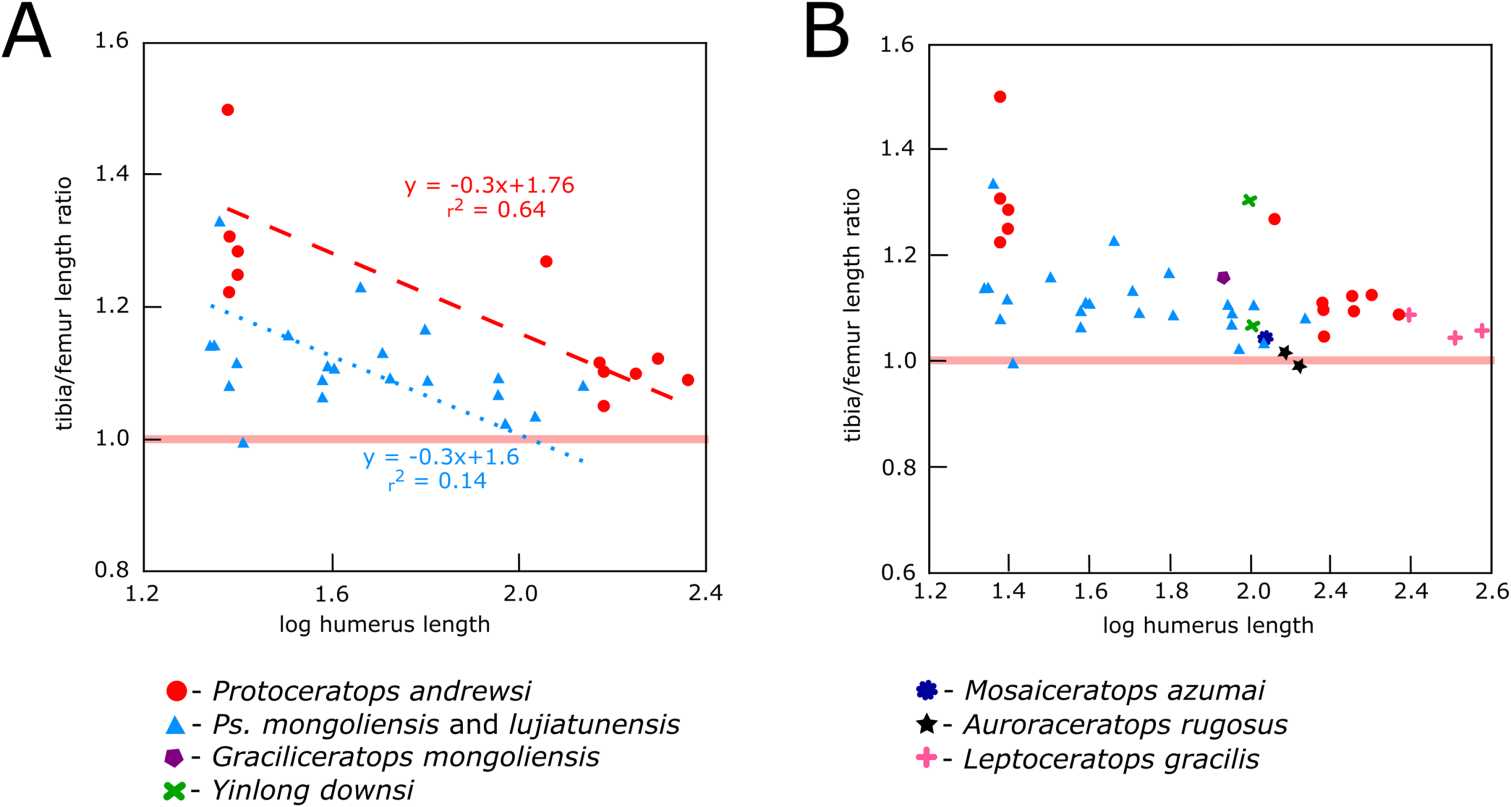
Relationship between tibia-to-femur length index and body size (using humeral length as a proxy). (A) *Protoceratops* and *Psittacosaurus* and (B) the same compared to other basal ceratopsians. Solid red line separates “bipeds,” which have the tibia longer than the femur from “quadrupeds,” where the proportions are reverse.

In *Protoceratops andrewsi* and *Psittacosaurus lujiatunensis* the tibia-to-femur length ratio overlaps considerably, showing in both species values typical of bipedal species (Figs. 20A and 20B). This ratio (with humeral length as a proxy for an animal’s age) shows a similar negative trend in ontogenetic change for *Protoceratops* than for *Psittacosaurus*. According to Maidment & Barrett (2012), a femur whose length exceeds that of the tibia is a strong indicator of quadrupedality and is not body-size dependent. It seems that during ontogeny of *Protoceratops andrewsi* the difference in length between the tibia and femur becomes smaller. In hatchlings the tibia is about 31% longer than femur (Fastovsky et al., 2011), in a small adult (AMNH 6471) the tibia is 10% longer than the femur, and in a large adult and old *Protoceratops andrewsi* the difference is even smaller, ca. 7% (AMNH 6417, 6416, and 6424). In *Psittacosaurus lujiatunensis*, an obvious biped for which numerous skeletons are known (see Zhao et al., 2013), the tibia also gets shorter in relation to the femur during ontogeny, and the length of the tibia exceeds that of the femur by 10% in hatchling, 13% in juvenile, 5% in subadult, and 8% in adult (Zhao et al., 2013; Hedrick et al., 2014), but the differences between the smallest specimens and the largest are not so pronounced as in *Protoceratops*. Even though the fact that the femur is longer than the tibia is regarded as strong morphological indicator for ornithischian quadrupedality, analysis of the proportions of the hind limb bones in *Psittacosaurus lujiatunensis* shows that some specimens of similar size have different tibia-to-femur length ratios, ranging from values strongly indicating bipedality to values indicating quadrupedality. The tibia is noticeably longer than the femur in the non-ceratopsid ceratopsian *Yinlong* (Xu et al., 2006). *Graciliceratops*, *Liaoceratops*, and *Ischioceratops* (He et al., 2015) also have high tibia-to-femur length ratios, comparable to those of *Psittacosaurus. Leptoceratops*, and *Mosaiceratops* (Zheng, Jin & Xu, 2015) have hind limb proportions comparable to those of adult *Protoceratops*, whereas in *Montanoceratops* and the early non-ceratopsid ceratopsian *Auroraceratops* (Morschhauser, 2012) the tibia is similar in length to the femur. Chinnery & Horner (2007) suggested bipedal posture for *Cerasinops*, based on the forelimb to hind limb length radio, similar to that of *Psittacosaurus*. This ratio can be, however, size-dependent in contrast to the tibia to femur length ratio (Maidment & Barrett, 2014). In *Cerasinops* the femur is longer than the tibia (Chinnery & Horner, 2007); the same holds for all Ceratopsidae, indicating obligate quadrupedality (Dodson, Forster & Sampson, 2004; Currie et al., 2016). However, the large disparity in the tibio-femoral ratio in *Psittacosaurus lujiatunensis* has to be noted (Fig. 20), indicating that the tibia-to-femur ratio may not be a decisive criterion for assessing walking posture.

## Discussion

Ceratopsians, like some other ornithischians (e.g., hadrosaurs), exhibit an evolutionary transition from bipedalism to quadrupedality (Maidment & Barrett, 2012, 2014). There are several skeletal characters related to quadrupedalism in Ornithischia (Maidment & Barrett, 2014): the presence of a craniolateral process on the ulna, a transversely broadened ilium, the femur longer than the tibia, a reduced fourth trochanter, and hoof-like manual unguals. We include in our functional analysis additional characters (the expansion of the scapular blade, proximal height of the olecranon process, and a straight femur in lateral view), considered as related to the animal’s posture (Chinnery, 2004a; Chinnery & Horner, 2007; Senter, 2007). In particular, they are related to the ability to put the forelimbs firmly on the ground and position them directly under the body, what allows for better support of the body and improved balance.

### Implications of the forelimb structure for gait mode and stance

The scapular blade is an attachment place of three major muscles: m. subscapularis medially, m. teres major and m. deltoideus scapularis laterally. The primary function of all these muscles (which insert near the proximal end of the humerus) is to stabilize the gleno-humeral joint. Moreover, the m. teres major draws the raised humerus downward and backward (Dilkes et al., 2012; Maidment & Barrett, 2014; Fearon & Varricchio, 2016). Enlargement of the scapular blade increases the area available for attachment of these muscles, presumably allowing them to become larger and stabilize the forelimb more effectively. Interestingly, Ceratopsidae have a narrow scapula bearing a large scapular spine laterally (Dodson, Forster & Sampson, 2004), which compensates for the lack of distal flaring.

Among non-ceratopsid ceratopsians, as well as in the ontogeny of *Protoceratops*, the distal end of scapular blade shows some variability in width. In juveniles and subadults of *Protoceratops andrewsi* the distal end of the scapula is narrow and similar to that of *Psittacosaurus* (although in this genus some variability was observed; see Hedrick et al., 2014), *Yinlong*, *Breviceratops*, and *Graciliceratops* (Fig. 4). Accordingly, the area of origination of m. teres major is not well expanded, indicating that this muscle became more important for *Protoceratops* in older age. On the other hand, in *Leptoceratops*, *Udanoceratops*, and *Auroraceratops*, the distal end is strongly expanded (Fig. 3), as in adult *Protoceratops*, suggesting strong scapular muscles, and indicating that m. teres major became more important for *Protoceratops* with age.

A set of characters relevant to the analysis of body posture is related to the range of movement at the glenoid joint. Senter (2007) suggested that a laterally expanded articular surface is typical of quadrupedal taxa, allowing them to move the humerus far forward. This pattern holds generally when the laterally narrow glenoid surface of *Psittacosaurus* and *Graciliceratops* (and young *Protoceratops*) is compared to the much more expanded glenoid cavity of *Leptoceratops*, *Auroraceratops*, and adult *Protoceratops*. However, subadult *Montanoceratops* and some members of Ceratopsidae (i.e., *Triceratops* and *Centrosaurus*) also have a laterally narrow glenoid, similar to bipedal *Psittacosaurus* or *Graciliceratops* (Fig. 4). Dodson & Farlow (1997) argue that the limited movement of the humerus due to the restricted glenoid articular surface may be, at least in part, compensated by a cartilage on the proximal end of the humerus. Nonetheless, the differences in the lateral expansion of the glenoid should be taken into consideration. We suggest that small bipedal ceratopsians have a limited lateral expansion of the glenoid cavity because the forelimbs were not used for dynamic movements, such as quick running (during escape). However, in the medium-sized ceratopsians (e.g., *Auroraceratops*, *Leptoceratops*, and adult *Protoceratops*), a larger range of motion of the humerus could have led to increased stride length and/or making easier turns when running, suggesting their quadrupedal mode of locomotion. In large non-ceratopsid ceratopsians (such as *Montanoceratops*) and Ceratopsidae, the glenoid is again narrow in the lateral view, which may be a result of the range of movements limited mostly to parasagittal plane (Thompson & Holmes, 2007), due to increased mass of the animal.

Another feature related to stabilizing the forelimb during quadrupedal gait is a high olecranon (Langer, Franca & Gabrieal, 2007) and craniolateral process of the ulna (Maidment & Barrett, 2014). *Yinlong*, *Psittacosaurus*, and *Breviceratops*, have a very low olecranon and craniolateral process; thus the elbow joint was not well-stabilized, and a quadrupedal gait was improbable in these genera. There is no information about the morphology of the olecranon process in juvenile *Protoceratops*; however, subadult ZPAL MgD-II/3 has a proximally extended process, similar to that of adult *Protoceratops*, Leptoceratopsidae, and *Auroraceratops*, which suggests a stable joint. The olecranon of ZPAL MgD-II/3 is medium in size in comparison to a very low olecranon in *Psittacosaurus*, and a very high one in Ceratopsidae (see Chinnery, 2004a; Dodson, Forster & Sampson, 2004 for the condition of the latter).

The quadrupedal stance and gait in some of non-ceratopsid neoceratopsians could be further inferred from a relative position of the ulna and radius; that is, whether they cross or lie in parallel (Fig. 9). This in turn may indicate whether the palms could be placed on the ground (the palmar side of the hand faces ventrally) or not (the palmar side faces medially), and thus determine whether the animal was bipedal or quadrupedal (Senter, 2007). In *Psittacosaurus* the radius does not cross the ulna (Senter, 2007), so the palms never faced the ground. On the other hand, the ulna and radius cross in *Protoceratops*, both in subadult ZPAL MgD-II/3 and in adult *Protoceratops*, and in this condition the palm faces caudally in the free hanging position or faces the ground when the animal stands quadrupedally. The same pattern generally holds: the bipeds have ulnae and radii that do not cross, but the respective bones cross in quadrupeds. *Leptoceratops*, which is considered here as mainly quadrupedal, and *Auroraceratops*, have a parallel radius and ulna. The bone arrangement in *Leptoceratops* is explained by structural adjustments to the hand and radius (a rolling proximal surface) that allow for an atypical position of the hand (Senter, 2007). Chinnery & Horner (2007) proposed that *Cerasinops* and *Udanoceratops* were bipedal because they have a distal ulna with a pronounced medial bend. Such distal bending can be even stronger in *Protoceratops* (Fig. 9E2) and is a result of the ulna and radius crossing (described above). Therefore, a bent ulna in *Cerasinops* and *Udanoceratops* suggests rather that these animals could place their palms down, and therefore supports their mainly quadrupedal gait.

The presence of flat unguals in the manus was proposed as a characteristic feature of quadrupedal ornithischians, explained as correlated with a heavy anterior part of the body, in particular a massive head (Maidment & Barrett, 2014). The manual and pedal unguals of ZPAL MgD-II/3 are pointed, not rounded, as commonly described for *Protoceratops* (Brown & Schlaikjer, 1940; You & Dodson, 2003; Maidment & Barrett, 2014). However, they are always flat, never claw-like, which is typical for obligatory bipeds, such as *Yinlong* or *Psittacosaurus*. Similar claw-like ungual morphology is also observed in Leptoceratopsidae (with the exception of *Udanoceratops*), *Auroraceratops*, *Mosaiceratops*, *Yamaceratops*, *Koreaceratops*, *Archaeoceratops*, *Graciliceratops*, and *Asiaceratops*.

On the other hand, flat and pointed (not rounded as in Ceratopsidae) unguals occur in *Udanoceratops*, *Protoceratops*, and *Bagaceratops* (Fig. 19). It was suggested that wide and flat unguals in *Bagaceratops* implied an aquatic mode of life, whereas those of *Protoceratops* and *Udanoceratops* pointed to an amphibious lifestyle (Tereshchenko, 2008). However, wide and flat unguals are usually associated with large body mass and tentatively correlated with the acquisition of a subunguligrade pes (Moreno, Carrano & Snyder, 2007). In the case of non-ceratopsid ceratopsians the occurrence of hoof-like unguals seems to be decoupled from the body size. *Protoceratops* and *Udanoceratops* co-occur in the Djadokhta Formation, while *Bagaceratops* is known from the Baruungoyot Formation. The Djadokhta and Baruungoyot formations are formed by deposits of structureless sandstones, with an inclusion of large-scale, aeolian cross-bed strata interpreted as sandstorm deposits or aeolian dunes (Jerzykiewicz, 2000). We suggest that the flat and wide unguals may have been an adaptation to moving on loose soil without sinking, and that they evolved convergently in at least some leptoceratopsids (e.g., *Udanoceratops*) and protoceratopsids.

### Implications of femoral structure for gait and stance in non-ceratopsid neoceratopsians

One of the most relevant features indicating quadrupedality in ornithischians is the pendent fourth trochanter (Maidment & Barrett, 2012, 2014). The fourth trochanter is the insertion of m. caudofemoralis brevis, which inserts on the ventral postacetabular blade, and m. caudofemoralis longus, which inserts on the caudal vertebrae. The first is supposed to be a femoral retractor (Maidment & Barrett, 2014), and the latter is considered the major retractor and medial rotator of the femur, providing the main locomotor power (Allen et al., 2010). Craniocaudal enlargement of the fourth trochanter, and therefore enlargement of m. caudofemoralis, were assumed to increase the mobility of the leg (Dilkes, 2001).

Positive allometric growth of the fourth trochanter in *Maiasaura* was previously proposed as an indicator of locomotor change during ontogeny (Dilkes, 2001). However, in *Alligator mississippiensis* it was proven experimentally not to play an essential role in locomotion (Allen et al., 2010). Nevertheless, in ornithischians a large and pendent fourth trochanter, indicating a strong m. caudofemoralis, played apparently more important role in locomotion than it does in crocodillians (Joneson et al. 2014), even in some species that otherwise appear well-adapted to quadrupedal locomotion.

Among Ceratopsia four different morphotypes of the fourth trochanter can be observed: (1) triangular and pendent, (2) parallelogram-shaped and pendent, (3) ridge-like, and (4) reduced (He et al., 2015). The fourth trochanter in *Protoceratops andrewsi* hatchlings (MPC-D 100/530) is placed low and protrudes as a slightly pendent plate (Fastovsky et al., 2011). In ZPAL MgD-II/3 it protrudes more caudally in comparison to the anatomy in juveniles, resembling the condition in adults (Brown & Schlaikjer, 1940; Maidment & Barrett, 2014). In *Psittacosaurus lujiatunensis* the fourth trochanter is not always pendent (Hedrick et al., 2014), so the reduction of this structure can occur in unambiguous bipeds (Maidment & Barrett, 2014). A triangular and pendent fourth trochanter occurs also in *Mosaiceratops*, *Auroraceratops*, *Archaeoceratops, Graciliceratops*, and *Cerasinops*. On the other hand, *Ischioceratops* and *Montanoceratops* have a parallelogram-shaped and pendent fourth trochanter, and *Protoceratops* and *Leptoceratops* have a ridge-like fourth trochanter.

Furthermore, it was suggested that the shape of the femur in lateral view (bowed or straight) can be informative on animal’s stance and gait mode. The bowed femur in lateral view was assumed a good correlate for ornithischian bipedality (Chinnery, 2004a; Yates et al., 2010). Maidment & Barrett (2014) argued that there is some correlation between large body size and a presence of a straight femur. However, exceptions may occur among non-ceratopsid ceratopsians, for example, a two-meter-long *Psittacosaurus sibiricus* (Averianov et al., 2006) has a bowed femur in lateral view, and the bone is straight in *Auroraceratops* of a similar size (Morschhauser, 2012), but otherwise expressed many skeletal features typical of quadrupeds.

Therefore, we decided to use the lateral shape of the femur as a possible discriminant feature of the locomotor habit. An arched femur in lateral view occurs in *Yinlong*, *Psittacosaurus*, *Mosaiceratops*, *Liaoceratops*, *Archaeoceratops*, *Graciliceratops*, and *Breviceratops*. It was reported that juvenile *Protoceratops* also have an arched femur (Fastovsky et al., 2011). Subadult and older individuals of *Protoceratops* have a straight femur in lateral view, similar to *Auroraceratops*, *Cerasinops*, *Ischioceratops*, *Montanoceratops*, and *Leptoceratops*, which indicates their adaptations to quadrupedal gait.

### Ontogenetic changes in the limbs of *Protoceratops*

There is almost no information about changes in locomotion throughout ontogeny in Ceratopsia. Among dinosaurs such ontogenetic transition was previously observed in three ornithopods: *Dryosaurus lettowvorbecki* (Heinrich, Ruff & Weishampel, 1993), *Maiasaura peeblesorum* (Dilkes, 2001), and *Iguanodon bernissartensis* (Norman, 1980). In these genera, the evidence for an ontogenetic locomotory shift comes from studies on the allometric growth of the limb bones, expressed in the overall lengthening of the forelimbs in relation to the hind limbs during ontogeny (Dilkes, 2001). However, Maidment & Barrett (2014) argued that it was difficult to determine whether the hind limb/forelimb ratio was (or was not) a good indicator of posture in ornithischians, and mentioned that there is a possibility of correlation with body size. Furthermore, the ratio of radius to humerus length, which are very similar for *Protoceratops andrewsi* and *Psittacosaurus lujiatunensis*, was also found to be uninformative for inferring any locomotor adaptations in Ornithischia as a whole, according to Maidment & Barrett (2014), in contrast to the tibia-to-femur length ratio. Bipedal ornithischians have a longer tibia than femur, but the reverse is true for quadrupedal species (Maidment & Barrett, 2014). According to the analyzed ratio (Fig. 20), *Protoceratops* still maintained limb proportions characteristic for bipeds, although it apparently spent most of its adult life as a quadruped. Even taking into account solely its head size, the head of *Protoceratops* was too heavy for a typical biped (for a detailed analysis of cranial ornamentation bearing on the position of the body mass center in ornithischians, see Maidment, Henderson & Barrett, 2014). The existing hind limb proportions can be therefore explained as an evolutionary heritage of the closest bipedal ancestors. Furthermore, this factor might have enabled juvenile and young adults to take a bipedal posture occasionally.

Taking a broader survey on the hind limb bones ratio among non-ceraratopsid neoceratopsians, the tibia is noticeably longer than the femur in *Yinlong* (Xu et al., 2006). *Graciliceratops*, *Liaoceratops*, and *Ischioceratops* (He et al., 2015), which have high tibia-to-femur length ratios, comparable to those of *Psittacosaurus* (Fig. 20B). *Leptoceratops* and *Mosaiceratops* (Zheng, Jin & Xu, 2015) have hind limb proportions comparable to those of adult *Protoceratops*, and in *Montanoceratops* and the early non-ceratopsid ceratopsian *Auroraceratops* (Morschhauser, 2012) the tibia is similar in length to the femur. Such adaptations in the last genus together with its forelimb morphology (including a high olecranon and strongly flared scapular blade) indicate rather strong adaptations to a quadrupedal gait.

## Conclusions

In this paper, we describe for the first time a complete appendicular skeleton of a subadult *Protoceratops andrewsi*, and compare its morphology and adaptations to those of non-ceratopsid ceratopsians. We scrutinized all of the characters relevant to locomotion and body posture. Overall, *Yinlong*, *Psittacosaurus*, *Graciliceratops*, *Mosaiceratops*, *Liaoceratops, Archaeoceratops*, and *Breviceratops* show many features indicative of bipedality. On the other hand, *Auroraceratops*, *Protoceratops*, and leptoceratopsids seem to be mainly quadrupedal, although their skeleton shows some traits which may indicate occasional ability to stand on their hind limbs.

The postcranial skeleton of *Protoceratops andrewsi*, especially that of mature specimens, shows a set of characters typical of Ceratopsia that presumably employed quadrupedal locomotion. Among these features are the flaring of the scapula blade, a wide glenoid cavity, a high olecranon process, and a crossed radius and ulna. These characters are present in most Neoceratopsia, including Ceratopsidae (where the flaring of the scapula blade is replaced by an eminent crest). Most of these postcranial characters in *Protoceratops* traced through ontogeny changed their state from those typical of bipedal ceratopsians (such as *Psittacosaurus*) to those characteristic of quadrupedal ones. This can be interpreted as an indication of facultative bipedalism in young individuals. In the case of the limb proportions, *Protoceratops* maintained a tibia-to-femur ratio typical of bipedal ceratopsians throughout its life.

The array of postcranial characters related to the mode of locomotion is distributed unevenly within the group of non-ceratopsid ceratopsians, which may point to the adaptive meaning of these characters. Some of them (e.g., the arching of the femur) are probably related to body mass, whereas other characters previously considered as body mass dependent (e.g., the shape of unguals) may be related to substrate conditions.

Our work provides a comparative base for skeletal morphology in non-ceratopsid neoceratopsians and stresses the importance of detailed anatomical studies for better understanding of functional morphology and early evolution of ceratopsian dinosaurs.

## Supplemental Information

10.7717/peerj.7324/supp-1 Supplemental Information 1 Supplemental Material: morphology and measurements.

10.7717/peerj.7324/supp-2 Supplemental Information 2 Right ilium fused with the sacral vertebrae of ZPAL MgD-II/3.

## Institutional Abbreviations

AMNH: American Museum of Natural History, New York, NY, USA
CASG-IG: Institute of Geology, Chinese Academy of Geological Sciences, Beijing, China
CMN: (formerly NMC), Canadian Museum of Nature, Ottawa, Ontario, Canada
GSGM: Gansu Geological Museum, Lanzhou, China
IGM: Institute of Geology, Ulaanbaatar, Mongolia
IVPP: Institute of Vertebrate Paleontology and Paleoanthropology, Chinese Academy of Sciences, Beijing, China
KIGAM VP: Korean Institute of Science and Mineral Resources, Vertebrate Paleontology, Daejeon, South Korea
MOR: Museum of the Rockies, Bozeman, MT, USA
MPCD-D: Mongolian Paleontological Center, Mongolian Academy of Sciences, Ulaanbaatar, Mongolia
NHMW: Natural History Museum Vienna, Vienna, Austria
PIN: Paleontological Institute, Russian Academy of Sciences, Moscow, Russia
PU: Princeton University, Princeton, NJ, USA
USNM: National Museum of United States, Smithsonian Institution, Washington D.C., USA
ZCDM: Zhucheng Dinosaur Museum, Zhucheng, China
ZPAL: Institute of Paleobiology, Polish Academy of Sciences, Warsaw, Poland.

## Additional Information and Declarations

### Data Availability

The following information was supplied regarding data availability: The specimens described here are stored at American Museum of Natural History, New York, NY, USA; Institute of Geology, Chinese Academy of Geological Sciences, Beijing, China; Gansu Geological Museum, Lanzhou, China; Institute of Geology, Ulaanbaatar, Mongolia; IVPP, Institute of Vertebrate Paleontology and Paleoanthropology, Chinese Academy of Sciences, Beijing, China; Korean Institute of Science and Mineral Resources, Vertebrate Paleontology, Daejeon, South Korea; Museum of the Rockies, Bozeman, MT, USA; Mongolian Paleontological Center, Mongolian Academy of Sciences, Ulaanbaatar, Mongolia; Natural History Museum Vienna, Vienna, Austria; Canadian Museum of Nature, Ottawa, Ontario, Canada; Paleontological Institute, Russian Academy of Sciences, Moscow, Russia; PU, Princeton University, Princeton, NJ, USA; National Museum of United States, Smithsonian Institution, Washington D.C., USA; Zhucheng Dinosaur Museum, Zhucheng, China; Institute of Paleobiology, Polish Academy of Sciences, Warsaw, Poland. The accession numbers for all 47 specimens are available in Table S1.
