# Supplementary material for: Appendicular skeleton of Protoceratops andrewsi (Dinosauria, Ornithischia): comparative morphology, ontogenetic changes, and the implications for non-ceratopsid ceratopsian locomotion
Source: PeerJ. 2019 Jul 22;7:e7324. doi: 10.7717/peerj.7324 (PMC6657679; doi:10.7717/peerj.7324)
Supplement: Supplemental Information 1 [file peerj-07-7324-s001.docx]

Supporting Information

**Appendicular skeleton of *Protoceratops andrewsi* (Dinosauria, Ornithischia): comparative morphology, ontogenetic changes and the implications for non-ceratopsid ceratopsian locomotion**

JUSTYNA SŁOWIAK, VICTOR S. TERESHCHENKO, and ŁUCJA FOSTOWICZ-FRELIK*

**Contents**

Table S1 (Comparative material) 2

Supplementary figures 6

Table S2 13

Table S3 14

*Corresponding author (lfost@twarda.pan.pl)

# Table S1 Comparative material

| Collection number | Locality  (formation) | Material |
| --- | --- | --- |
| ***Protoceratops* *andrewsi*** | | |
| AMNH FR6408 | ‘Shabarakh Usu’ =  Bayn Dzak, Mongolia  (Djadokhta Formation) | Both sternal plates, right incomplete forelimb including scapula, humerus, ulna and radius |
| AMNH FR6417 |  | Complete mounted skeleton |
| AMNH FR6418 |  | Right forelimb without manus and left forelimb including proximal humerus, complete ulna, radius, and fragmentary metacarpals. Pelvis include left and right ilium, ischium, and right pubis. Incomplete left femur is also preserved. |
| AMNH FR6419 |  | Right forelimb includes: scapula, humerus ulna and radius. Only left scapulocoracoid is preserved from the left forelimb. |
| AMNH FR6424 |  | Both ilia are preserved, and also both femora, tibiae with astragalus and calcaneum, fibulae, and right fragmentary metatarsals. |
| AMNH FR6426 |  | Right scapula, humerus, ulna and radius. Right ischium and femur |
| AMNH FR6453 |  | Both ilia are preserved and left ischium. Right femur, tibia, and fibula are present. Fragmentary left metatarsals and pes unguals |
| AMNH FR6466 |  | Left pelvic bones are preserved and right fragmentary pubis. |
| AMNH FR6467 |  | Complete mounted skeleton |
| AMNH FR6470 |  | Left pelvis bones |
| AMNH FR6471 |  | Right scapulocoracoid |
| AMNH FR6475 |  | Right ilium, femur and fragmentary metatarsals. Left tibia with fibula |
| AMNH FR6481 |  | Both ilia and femora. Right tibia and fibula |
| AMNH FR6494 |  | Both ischia, right tibia, fibula and fragmentary pes |
| AMNH FR6636 |  | Right scapula and humerus, left humerus and radius, left ilium, left and right femur, left tibia and fibula |
| NHMW 2015/0404/0001 | Bayn Dzak (?), Mongolia  (Djadokhta Formation)  Specimen bought by NHMW | Incomplete skull, fragmentary right scapula, complete left scapula, proximal right humerus, fragmentary right and left radius. Incomplete pelvis (fragments of ilium and ischium), incomplete left femur, and both left and right fragmentary tibia and fibula. Fragmentary pes and manus. Incomplete vertebral column |
| PIN 3143/6 | Toogreek, Mongolia  (Djadokhta Formation) | Both scapulae, ilium |
| PIN 3143/5 |  | Almost complete skeleton |
| PIN 3143/7 |  | Almost complete skeleton |
| PIN 3143/9 |  | Both coracoids, scapulae, humeri, pelvis, left femur, and both tibiae |
| PIN 3143/15 |  | Both femora and tibiae. Right incomplete pes |
| PIN 3143/4 |  | Left coracoid, both scapulae, left humerus, both ulnae and radii. Left femur, both tibiae and fibulae, right fragmentary metatarsals |
| ZPAL MgD-II/11 | Bayn Dzak, Mongolia  (Djadokhta Formation) | Incomplete left hind limb and pubis. Dorsal vertebrae |
| ZPAL MgD-II/16 |  | Left pelvic bones and left femur |
| ZPAL MgD-II/35 | Toogreek, Mongolia  (Djadokhta Formation) | Almost complete skeleton |
| ZPAL MgD-II/407 | Bayn Dzak, Mongolia  (Djadokhta Formation) | Distal femur |
| ZPAL MgD-II/408 |  | Incomplete tibia |
| ***Bagaceratops* *rozhdestvenskyi*** | | |
| ZPAL MgD-I/142 | ‘Khermeen Tsav’ = Hermiin Tsav, Mongolia  Baruungoyot Formation | Proximal right femur |
| ZPAL MgD-I/155 |  | Right pelvis with articulated sacrum |
| ZPAL MgD-I/320 |  | Articulated right pes with fragmentary tibia and fibula (?) |
| ***Breviceratops* *kozlowskii*** | | |
| ZPAL MgD-I/117 | Khulsan, Mongolia  Baruungoyot Formation | Skull with almost complete postcranial skeleton |
| ***Graciliceratops* *mongoliensis*** | | |
| ZPAL MgD-I/156 | Sheeregeen Gashoon, Mongolia  (‘Sheeregeen Gashoon Formation’ = Baynshire Formation) | Fragmentary skull and left forelimb without manus, and complete right hind limb Fragmentary pelvic elements and incomplete vertebral column |
| ***Leptoceratops* *gracilis*** | | |
| AMNH FR5205 | Tolman Ferry, Alberta  (Edmonton Group) | Right scapulocoracoid, humerus, radius, and both ulnae. Right incomplete femur, and tibia with astragalus. Pes ungual |
| AMNH FR5208 |  | Right scapulocoracoid and left pes elements |
| ***Montanoceratops* *cerorhynchus*** | | |
| AMNH FR5464 | Buffalo Lake, Montana  (St. Mary Formation) | Both ilia, ischia, and right pubis. Both femora, left tibia, fibula, astagalus and calcaneum. Pes unguals |
| ***Psittacosaurus* *mongoliensis*** | | |
| AMNH FR6254 | Oshih Basin, Mongolia  (Oshih Formation) | Complete articulated skeleton |
| AMNH FR6260 |  | Fragmentary forelimbs and hind limbs |
| AMNH FR6534 |  | Right femur and ilium |
| AMNH FR6535 |  | Rib cage with dorsal vertebrae, ribs and both scapulocoracoids |
| AMNH FR6537 |  | Left scapula, humerus, fragmentary femur and ilium |
| AMNH FR6538 |  | Left femur and fragmentary pes. |
| AMNH FR6541 |  | Right coracoid, both fragmentary ilia, and femora, right tibia, and calcaneum |
| AMNH FR6544 |  | Right scapulocoracoid, and both humeri, femora, tibiae, and right fibula, astagalus, and calcaneum |
| NHMW 1998z0064/0001 | Specimen bought by NHMW | Right scapulocoracoid, left humerus. Left ilium, both femora, incomplete tibiae and fibulae |
| CV 738 | Toushan, Shandong Province, China  (Qingshan Formation) | Almost complete articulated skeleton (cast) |
| ***Psittacosaurus neimongoliensis*** | | |
| IVPP 12-0888-2  (IVPP RV96001) | Yangpo (?), Dongsheng, Inner Mongolia  (Ejinhoro Formation) | Complete skeleton |
| ***Psittacosaurus xinjiangensis*** | | |
| IVPP V7698 | Doulusshan, Junggar Basin, Xinjiang Uygur Autonomous Region  (Tugulo Group) | Complete articulated skeleton |

# Supplementary figures


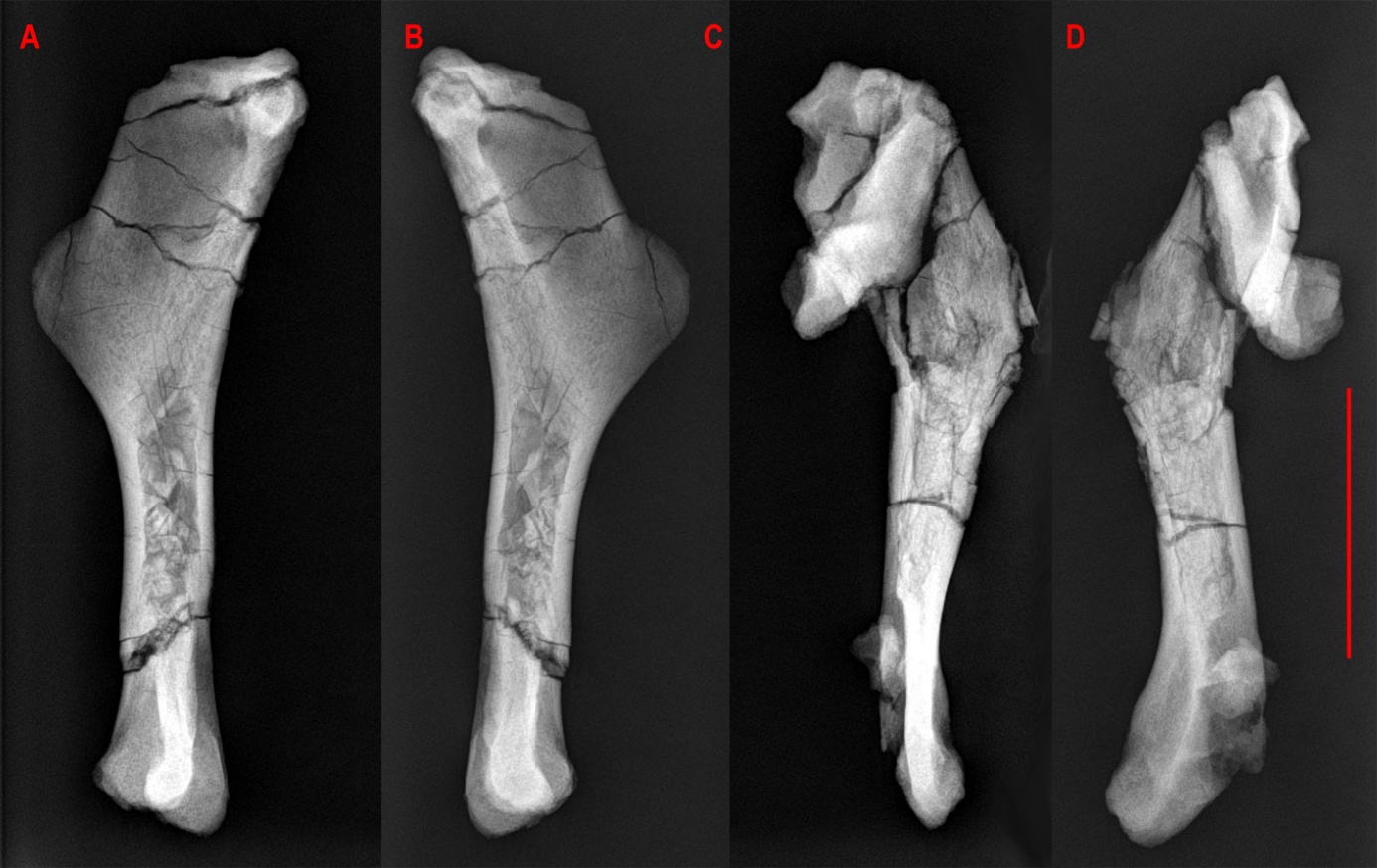


**Fig. S1.** X-ray image of left (**A**, **B**) and right (**C**, **D**) humeri of ZPAL MgD-II/3, showing the cleavage planes and bone crushing; **A**, **C**, lateral views, **B**, **D**, medial views. Scale bar 4 cm.


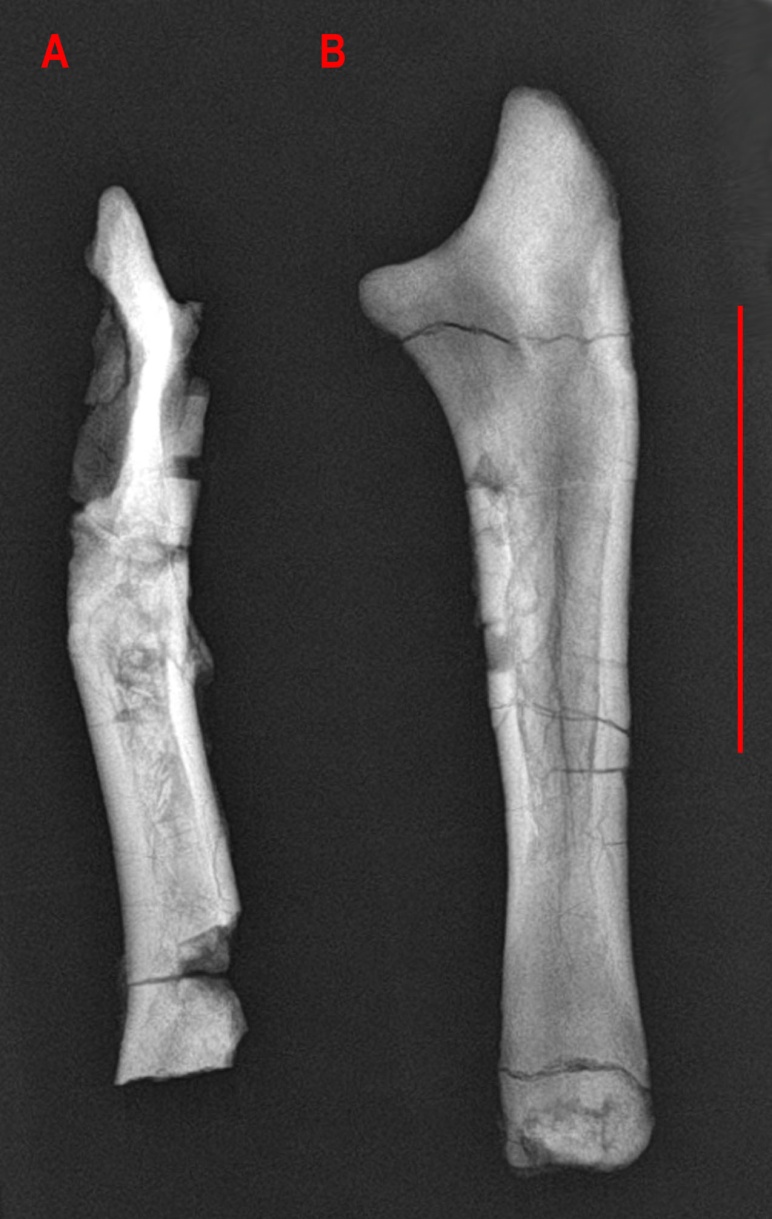


**Fig. S2.** X-ray image of right (**A**) and left (**B**) ulnae of ZPAL MgD-II/3. **A**, a poorly preserved right ulna; **B**, a well-preserved left ulna; both in lateral view. Scale bar: 4 cm.


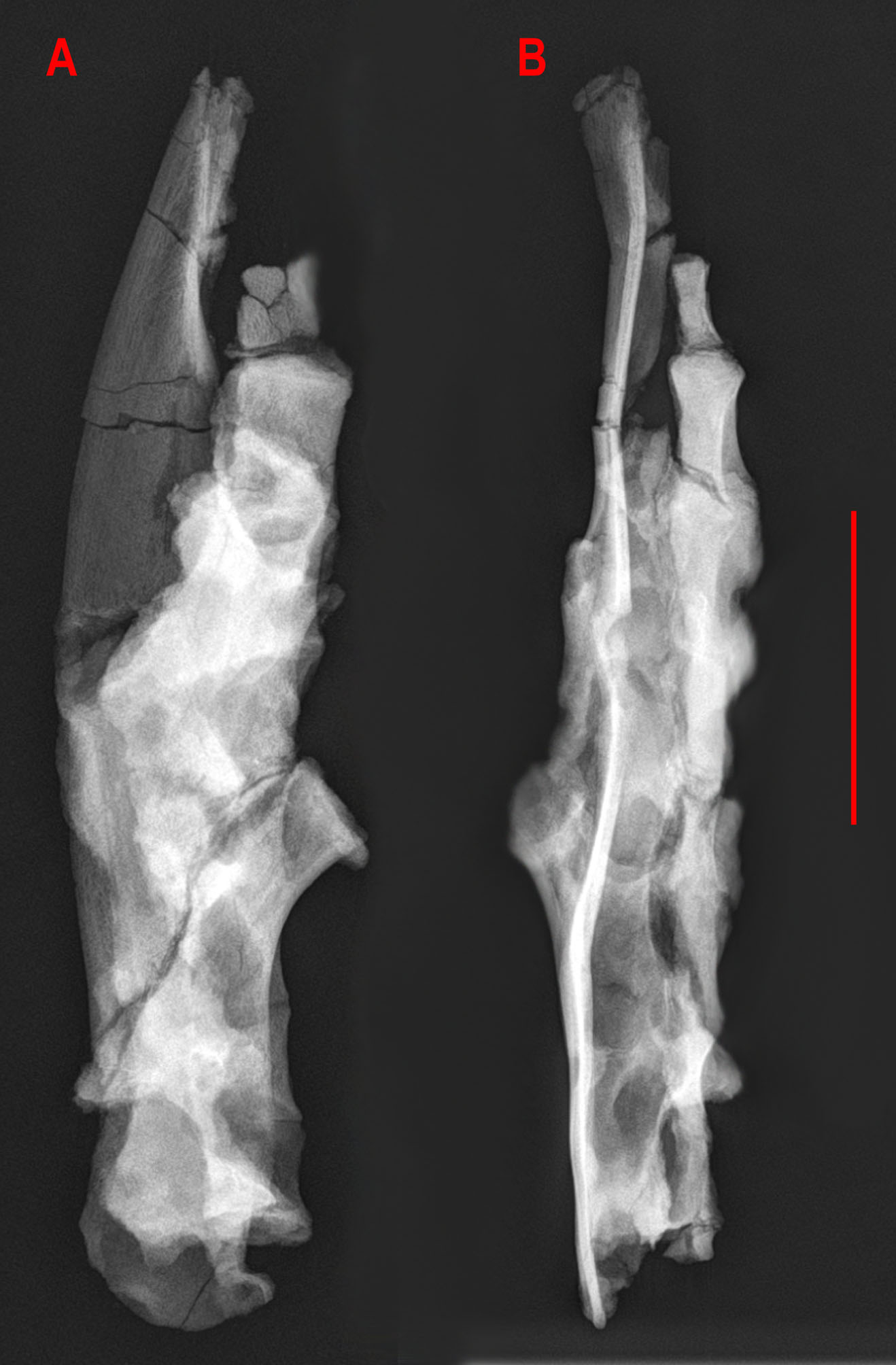


**Fig. S3.** X-ray image of a right ilium fused with the sacral vertebrae of ZPAL MgD-II/3. **A**, medial, and **B**, dorsal views. Scale bar 4 cm.


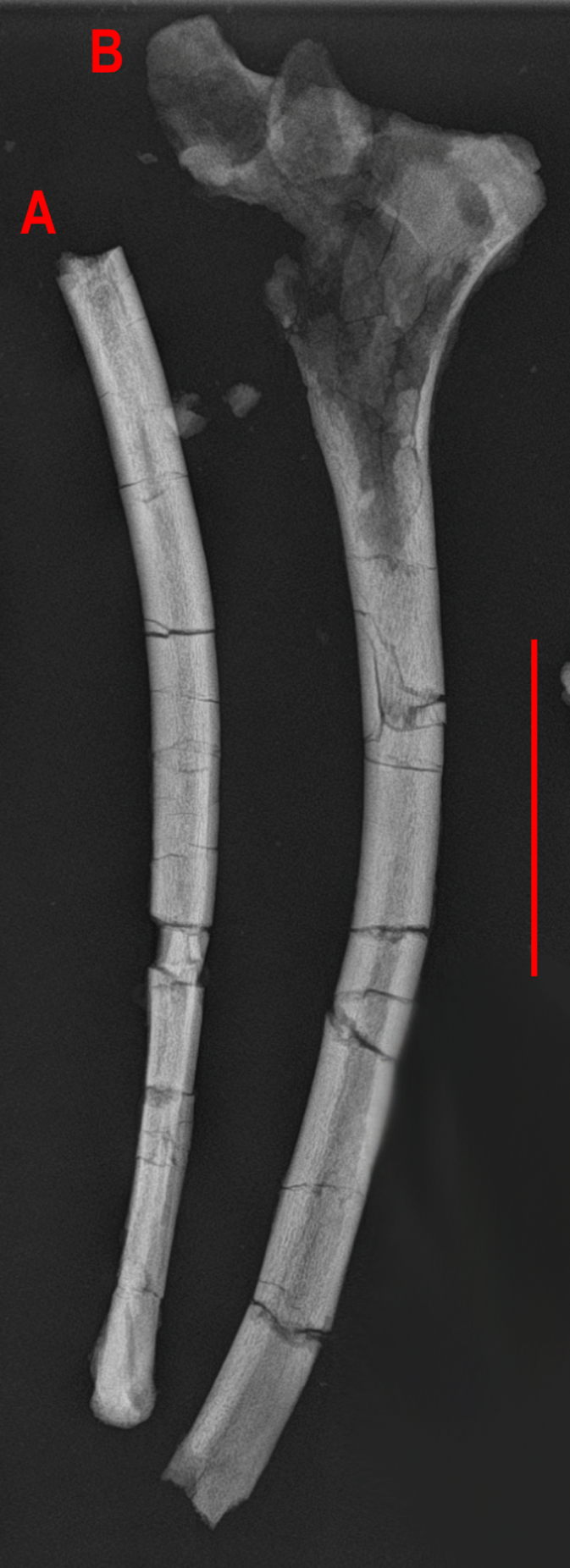


**Fig. S4.** **X-ray image** of right (**A**) and left (**B**) ischium of ZPAL MgD-II/3. **A**, ventral; **B**, medial views. Note strongly damaged proximal part. Scale bar 4 cm.


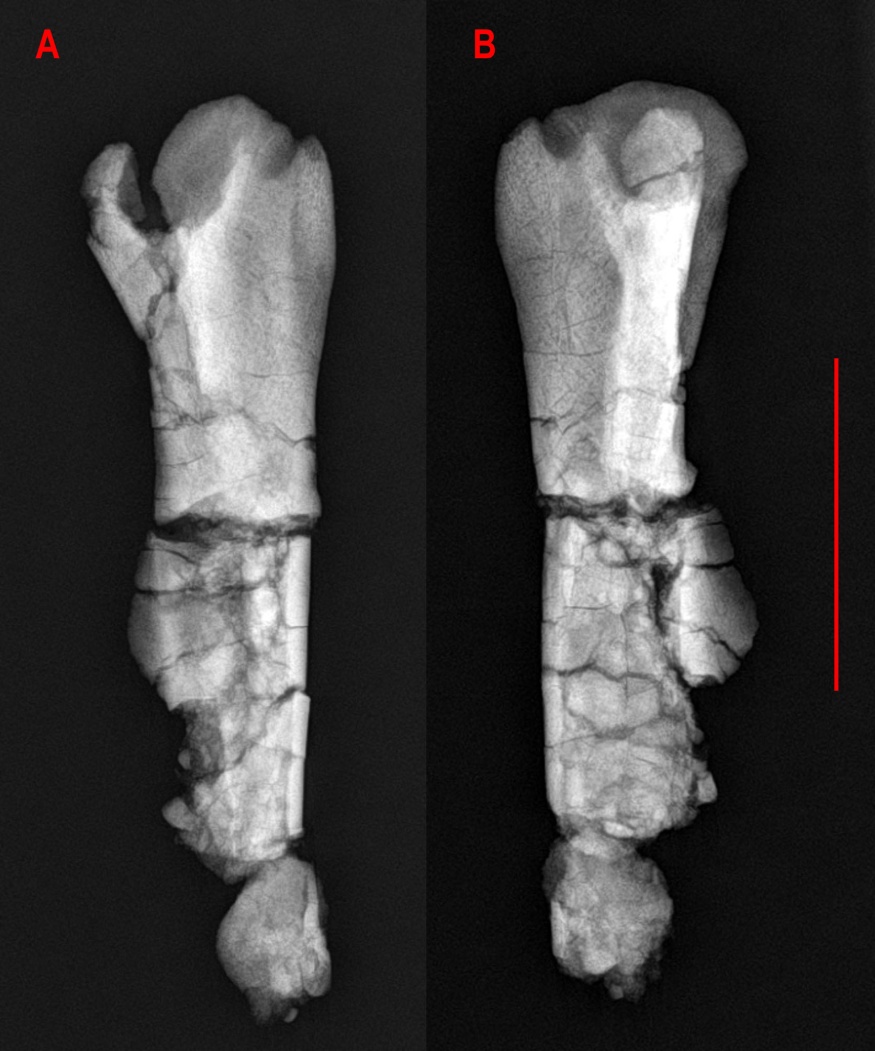


**Fig. S5.** X-ray image of a right femur of ZPAL MgD-II/3. **A**, cranio-lateral, and **B**, medial views. Note damaged shaft. Scale bar 4 cm.


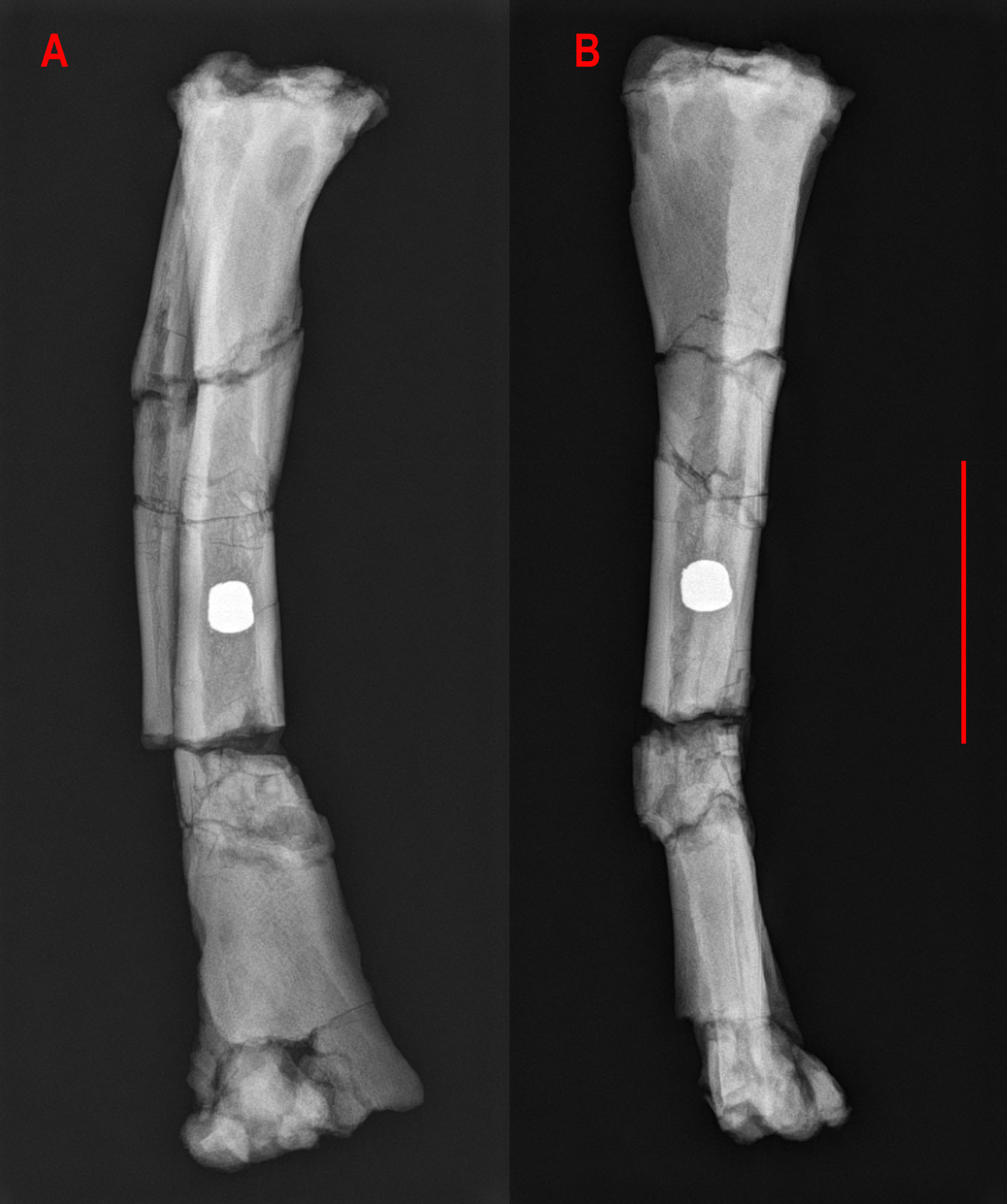


**Fig. S6.** X-ray image of right tibia and fibula of ZPAL MgD-II/3. **A**, cranial, and **B**, medial view. Note damaged shaft. In proximal part of the bone, some larval borings are visible as an elongated shadow. Inside the shaft, an unidentified dense object was found. Scale bar 4 cm.


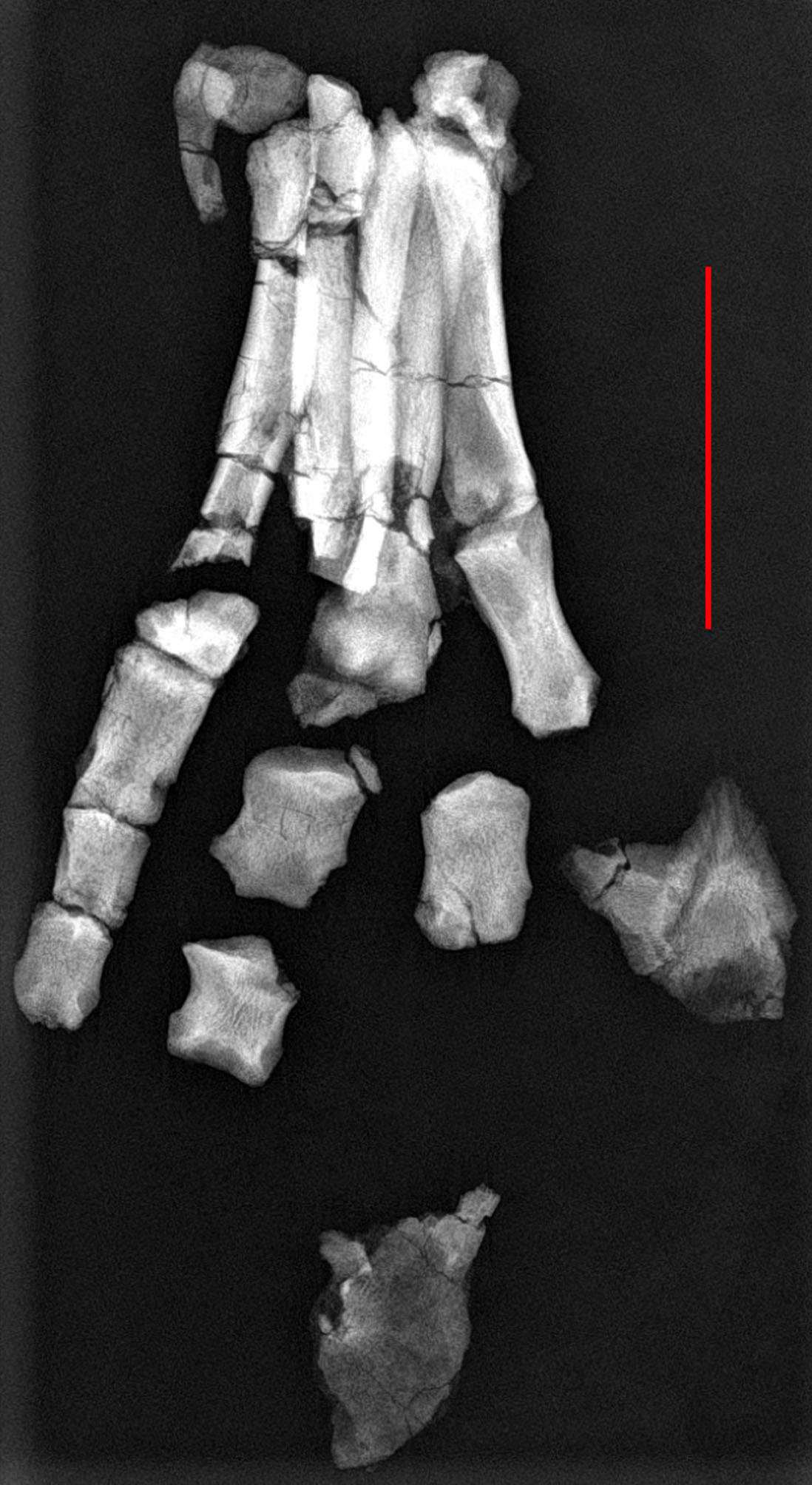


**Fig. S7.** X-ray image of right (**A**) and left (**B**) ischia of ZPAL MgD-II/3. Scale bar 4 cm.

**Table S2 Femur measurements (in mm)**

| **Species** | **Catalog number** | **Total length** | **Length of femur distal to fourth trochanter** | **Fourth trochanter length** |
| --- | --- | --- | --- | --- |
| *Protoceratops*  *andrewsi* | MPC-D 100/530 | 25.0 | 14.0 | 4.0 |
|  | ZPAL MgD-II/3 | 142.8 |  | 25.7 |
|  | AMNH 6418 | 183.7 | 51.1 | 35 |
|  | AMNH 6424 | 248.0 | 108.0 | 55.5 |
|  | AMNH 6417 | 221.0 | 113.0 | 32.7 |
|  | PIN 3143/5 L | 208.0 | 109.3 | 28.0 |
|  | PIN 3143/5 R | 216.0 | 107.1 | 30.0 |
|  | PIN 3143/7 R | 215.0 | 96.5 | 25.3 |
|  | PIN 3143/9 | 249.0 | 114.0 | 38.3 |
|  | PIN 3143/4 | 265.0 | 130.0 | 43.2 |
|  | PIN 3142/15 | 234.0 | 116.0 | 34.2 |
|  |  |  |  |  |
| *Psittacosaurus*  *mongoliensis* | NHMW 1998z0064/0001 | 114.0 | 56.0 | 17.0 |
|  | IVPP CV738 | 95.7 | 46.0 | 14.0 |
|  | AMNH 6541 | 178.8 | 79.0 | 34.3 |
|  | IVPP RV96001 | 163.0 | 84.0 | 24.0 |

**Table S3 Long bones length (in mm)**

| **Species** | **Catalog number** | **Radius** | **Humerus** | **Tibia** | **Femur** | **Ref.** |
| --- | --- | --- | --- | --- | --- | --- |
| *Protoceratops andrewsi* | MPC-D 100/530-6 | 22.0 | 24.0 | 36.0 | 24.0 | Fastovsky et al. 2011 |
|  | MPC-D 100/530-5 | 20.0 | 22.0 |  | 25.0 | Fastovsky et al. 2011 |
|  | MPC-D 100/530-9 | 19.0 | 24.0 | 34.0 | 26.0 | Fastovsky et al. 2011 |
|  | MPC-D 100/530-11 |  |  | 34.0 | 26.0 | Fastovsky et al. 2011 |
|  | MPC-D 100/530-3 | 19.0 | 24.0 | 33.0 | 27.0 | Fastovsky et al. 2011 |
|  | MPC-D 100/530-4 | 18.0 |  | 36.0 | 27.0 | Fastovsky et al. 2011 |
|  | MPC-D 100/530-7 | 19.0 | 25.0 | 35.0 | 28.0 | Fastovsky et al. 2011 |
|  | MPC-D 100/530-2 | 20.0 | 25.0 | 36.0 | 28.0 | Fastovsky et al. 2011 |
|  | AMNH 6419 | 42.0 | 550 |  |  |  |
|  | ZPAL MgD-II/3 | 75.0 | 105.0 | 149.0 |  |  |
|  | IVPP uncataloged | 80.0 | 115.0 | 165.0 | 130.0 | Maidment and Barrett 2014 |
|  | PIN 614-42/1 |  |  |  | 164.2 |  |
|  | AMNH 6453 |  |  | 198.0 | 170.0 |  |
|  | AMNH 6471 | 112.0 | 152.0 | 208.0 | 189.0 |  |
|  | CM 9185 | 107.0 | 153.0 | 203.5 | 194.0 | Chinnery 2004a |
|  | AMNH 6418 | 108.0 | 177.0 |  |  |  |
|  | PIN 3143/7 |  |  | 210.0 | 215.0 |  |
|  | PIN 3143/5 | 127.0 | 180.0 | 228.0 | 218.1 |  |
|  | AMNH 6417 |  |  | 241.0 | 221.0 |  |
|  | AMNH 6416 |  |  | 241.0 | 226.0 |  |
|  | PIN 3143/15 |  |  | 260.0 | 234.0 |  |
|  | AMNH 6424 | 135.0 | 210.0 | 270.0 | 248.0 |  |
|  | PIN 3143/9 |  | 200.0 | 280.0 | 249.0 |  |
|  | PIN 3143/4 |  |  |  | 265.0 |  |
|  |  |  |  |  |  |  |
| *Yinlong downsi* | IVPP V14530 | 51.0 | 99.0 |  | 155.0 | Maidment and Barrett 2014 |
|  | IVPP 18684 | 60.2 | 101.4 | 179.0 | 167.9 |  |
|  | IVPP WCW-O6A-38 |  | 100.0 | 215.0 | 165.0 | Maidment and Barrett 2014 |
|  |  |  |  |  |  |  |
| *Psittacosaurus lujiatunensis* | IVPP V16902.1 | 18.0 | 22.0 | 25.0 | 22.0 | Zhao et al. 2013 |
|  | IVPP V16902.2 | 20.0 | 24.0 | 27.0 | 25.0 | Zhao et al. 2013 |
|  | IVPP V16902.3 | 21.0 | 25.0 | 29.0 | 26.0 | Zhao et al. 2013 |
|  | DMNH D2156-4 | 21.7 | 23.0 | 39.9 | 29.9 | Hedrick et al. 2014 |
|  | DMNH D2156-9 | 20.2 | 22.4 | 35.2 | 31 | Hedrick et al. 2014 |
|  | DMNH D2156-17 |  |  | 40.3 | 33 | Hedrick et al. 2014 |
|  | DMNH D2156-5 |  | 25.8 | 35.5 | 35.7 | Zhao et al. 2013 |
|  | DMNH D2156-26 |  |  | 37.0 | 34.8 | Hedrick et al. 2014 |
|  | DMNH D2156-12 |  |  | 42.8 | 35.8 | Hedrick et al. 2014 |
|  | ELDM V1037 | 26.0 | 32.0 | 44.0 | 38.0 | Zhao et al. 2013 |
|  | ELDM V1038.21 | 30.0 | 38.0 | 48.0 | 44.0 | Zhao et al. 2013 |
|  | ELDM V1038.15 | 31.0 | 39.0 | 51.0 | 46.0 | Zhao et al. 2013 |
|  | ELDM V1038 | 38.0 | 38.0 | 50.0 | 47.0 | Zhao et al. 2013 |
|  | ELDM V1038.11 | 31.0 | 40.0 | 52.0 | 47.0 | Zhao et al. 2013 |
|  | IVPP V14341.5 | 32.0 | 44.0 |  |  | Maidment and Barrett 2014 |
|  | IVPP V14341.4 | 27.0 | 46.0 | 65.0 | 53.0 | Maidment and Barrett 2014 |
|  | IVPP V14341.2 |  | 51.0 | 69.0 | 61.0 | Maidment and Barrett 2014 |
|  | IVPP V14341.3 | 34.0 | 53.0 | 72.0 | 66.0 | Maidment and Barrett 2014 |
|  | IVPP V14341.1 | 41.0 | 63.0 | 84.0 | 72.0 | Maidment and Barrett 2014 |
|  | IVPP V14342 | 41.0 | 64.0 | 88.0 | 81.0 | Zhao et al. 2013 |
|  | IVPP V14748 | 54.0 | 90.0 | 119.0 | 109.0 | Zhao et al. 2013 |
|  | IVPP V14749 | 56.0 | 90.0 | 125.0 | 117.0 | Zhao et al. 2013 |
|  | IVPP V18343 | 64. 0 | 94.0 | 135.0 | 132.0 | Zhao et al. 2013 |
|  | IVPP V18344 | 75.0 | 108.0 | 150.0 | 145.0 | Zhao et al. 2013 |
|  | IVPP V12716 | 85.0 | 137.0 | 175.0 | 162.0 | Zhao et al. 2013 |
|  |  |  |  |  |  |  |
| *Psittacosaurus mongoliensis* | AMNH 6538 |  |  | 128.6 | 113.4 |  |
|  | NHMW 1998z0064/0001 | 87.0 | 102.0 | 126.0 | 114.0 |  |
|  | AMNH 6253 | 80.0 | 88.0 | 167.0 | 151.0 |  |
|  | AMNH 6254 |  |  | 181.0 | 159.0 |  |
|  | AMNH 6454 |  |  | 179.0 | 162.0 |  |
|  | AMNH 6541 |  |  | 189.5 | 178.8 |  |
|  | GI SPS 100/606 |  | 83.0 | 100.0 | 99.0 | Sereno 1987 |
|  | IVPP CV738 |  |  | 102.6 | 95.7 |  |
|  |  |  |  |  |  |  |
| *Graciliceratops mongoliensis* | ZPAL-MgDI/156 | 60,00 | 86,00 | 110,00 | 95.00 |  |
|  |  |  |  |  |  |  |
| *Mosaiceratops azumai* |  | 89.0 | 108.0 | 143.0 | 137.0 |  |
|  |  |  |  |  |  |  |
| *Auroraceratops rugosus* | GJ (07)9-02/2-12 | 75.1 | 122.1 | 158.0 | 155.6 | Morschhauser 2012 |
|  | GJ (08)22 |  | 132.6 | 161.8 | 163.2 | Morschhauser 2012 |
|  |  |  |  |  |  |  |
| *Leptoceratops gracilis* | USNM 13863 |  |  | 200.0 |  | Ostrom 1978, but referred to *Cerasinops* *hodgskissi* by Chinnery and Horner (2007) |
|  | NMC 8887 | 115.0 | 185.0 | 240.0 | 230.0 | Sternberg 1951 |
|  | NMC 8888 | 137.0 | 255.0 | 280.0 | 265.0 | Sternberg 1951 |
|  | NMC 8889 | 163.0 | 248.0 | 311.0 | 286.0 | Chinnery 2004a |
|  | AMNH 5205 | 167 | 290.0 |  |  |  |
