# Supplementary figures and images for: Appendicular skeleton of Protoceratops andrewsi (Dinosauria, Ornithischia): comparative morphology, ontogenetic changes, and the implications for non-ceratopsid ceratopsian locomotion
Source: PeerJ. 2019 Jul 22;7:e7324. doi: 10.7717/peerj.7324 (PMC6657679; doi:10.7717/peerj.7324)

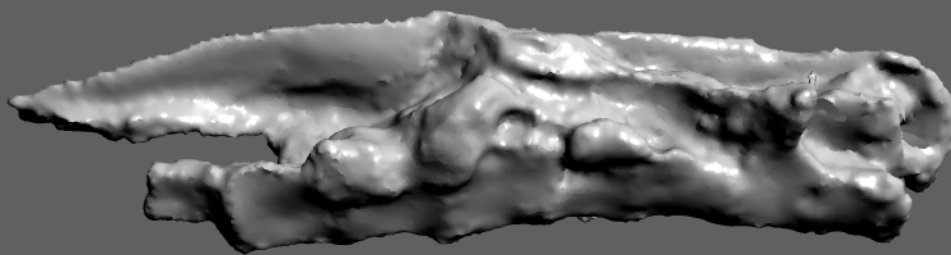

Interactive 3D model of the ilium and sacrum of *Protoceratops anrewsi* ZPAL MgD-II/3

Supplement: Supplemental Information 2 [file peerj-07-7324-s002.pdf]
